# Responsiveness to feedback as a personal trait

**DOI:** 10.1007/s11166-018-9277-3

**Published:** 2018-05-10

**Authors:** Thomas Buser, Leonie Gerhards, Joël van der Weele

**Affiliations:** 1 Tinbergen Institute, University of Amsterdam, Amsterdam, Netherlands; 2 University of Hamburg, Hamburg, Germany; 3 CREED, Tinbergen Institute, University of Amsterdam, Amsterdam, Netherlands

**Keywords:** Bayesian updating, Feedback, Confidence, Identity, Competitive behavior, C91, C93, D83

## Abstract

We investigate individual heterogeneity in the tendency to under-respond to feedback (“conservatism”) and to respond more strongly to positive compared to negative feedback (“asymmetry”). We elicit beliefs about relative performance after repeated rounds of feedback across a series of cognitive tests. Relative to a Bayesian benchmark, we find that subjects update on average conservatively but not asymmetrically. We define individual measures of conservatism and asymmetry relative to the average subject, and show that these measures explain an important part of the variation in beliefs and competition entry decisions. Relative conservatism is correlated across tasks and predicts competition entry both independently of beliefs and by influencing beliefs, suggesting it can be considered a personal trait. Relative asymmetry is less stable across tasks, but predicts competition entry by increasing self-confidence. Ego-relevance of the task correlates with relative conservatism but not relative asymmetry.

**Electronic supplementary material:**

The online version of this article (10.1007/s11166-018-9277-3) contains supplementary material, which is available to authorized users.

## Introduction

The ability and willingness to take into account feedback on individual performance can influence important choices in life. Mistakes in incorporating feedback may lead to the formation of under- or overconfident beliefs that are known to be associated with inferior decisions. Reflecting the importance of the topic, there is a substantial literature in psychology and economics on Bayesian updating and the role of feedback in belief formation, which we review in more detail below. This literature shows that people are generally “conservative”, i.e. they are less responsive to noisy feedback than Bayesian theory prescribes. Möbius et al. (2014, henceforth MNNR) also suggest that people update in an “asymmetric” way about ego-relevant variables, placing more weight on positive than on negative feedback about their own ability.

We investigate heterogeneity in feedback responsiveness, and ask whether it can be considered a personal trait that explains economic decisions. In our experiment, we measure how participants update their beliefs about their relative performance on three cognitive tasks. The tasks require three distinct cognitive capabilities, namely verbal skills, calculation skills and pattern recognition. We recruited subjects from different study backgrounds, creating natural variation in the relevance of the three tasks for the identity or ego of different subjects. The feedback structure is inspired by MNNR, and consists of six consecutive noisy signals after each task about the likelihood that they scored in the top half of their reference. We thus elicit six belief updates on each task, which allows us to construct measures of conservatism and asymmetry for each subject.

Our experiment generates a number of important new insights on feedback responsiveness. We first show that on average, people do not update like Bayesians. About one quarter of updates are zero, and ten percent go in the wrong direction. The remainder of the updates are too conservative, and unlike a true Bayesian, subjects don’t condition the size of the belief change on the prior belief.

Our main analysis concerns the heterogeneity of updating between individuals. To this end, we define measures of relative individual conservatism and asymmetry, based on individual deviation from the average updates of all subjects with similar priors. We find that relative conservatism is correlated across tasks and can be considered a personal trait. By contrast, relative asymmetry does not appear to be a stable trait of individuals. Using within-subject variation, we find that the ego-relevance of a task leads to higher initial beliefs about being in the top half, and leads subjects to update more conservatively but not more asymmetrically.

When it comes to the impact of heterogeneity, we show that differences in feedback responsiveness are important in explaining both beliefs and decisions. Variation in feedback responsiveness between individuals explains 21% of variation in post-feedback beliefs, controlling for the content of the feedback. A standard deviation increase in relative conservatism raises (lowers) beliefs for individuals with many bad (good) signals by 10 percentage points on average. A standard deviation in relative asymmetry raises beliefs for any feedback, and by up to 22 percentage points for subjects with a similar number of positive and negative feedback signals.

Moreover, feedback responsiveness explains subjects’ willingness to compete with others, a decision that is predictive of career choices outside of the lab (see the literature review below). We measure willingness to compete on a final task, composed from exercises similar to each of the previous tasks. Subjects choose whether they want to get paid on the basis of an individual piece rate, or on the basis of a winner-takes-all competition against another subject. We find that relative conservatism predicts entry into competition both through influencing final beliefs and independently of beliefs. Relative asymmetry also predicts entry by raising final beliefs. Thus, being more conservative and asymmetric is good for high-performing subjects with an expected gain from competition, and bad for the remaining subjects.

To our knowledge, this paper provides the most in-depth investigation so far of the importance of individual feedback responsiveness for beliefs about personal ability and economic decisions. Our findings suggest that individual differences in conservatism and, to a lesser degree, asymmetry help explain differences in self-confidence and willingness to compete. In the conclusion, we provide a range of domains in which we expect these attributes to affect people’s decisions and discuss how our study can help to reduce the negative effects of faulty updating.

## Literature

A sizable literature in psychology on belief updating has identified that people are generally “conservative”, meaning they are less responsive to noisy feedback than Bayesian theory suggests (Slovic and Lichtenstein 1971; Fischhoff and Beyth-Marom 1983). More recent evidence shows that when feedback is relevant to the ego or identity of experimental participants, they tend to update differently (Möbius et al. 2014; Eil and Rao 2011; Ertac 2011; Grossman and Owens 2012). These studies provide a link between updating behavior and overconfidence, as well as to a large literature on self-serving or ego biases in information processes (see e.g. Kunda 1990).

More specifically, MNNR use an experimental framework with a binary signal and state space that allows explicit comparison to the Bayesian update. They find evidence for asymmetric updating on ego-relevant tasks, showing that subjects place more weight on positive than on negative feedback. Furthermore, there is neurological and behavioral evidence that subjects react more strongly to successes than to failures in sequential learning problems (Lefebvre et al. 2017), and update asymmetrically about the possibility of negative life events happening to them (Sharot et al. 2011). These papers are part of a wider discussion about the existence of a general “optimism bias” (Shah et al. 2016; Marks and Baines 2017).

At the same time, Schwardmann and van der Weele (2016), Coutts (2018), Barron (2016), and Gotthard-Real (2017) do not find asymmetry, using variations of the MNNR framework that differ in the prior likelihood of events and whether the stakes in the outcome are monetary or ego-related. The first two studies even find a tendency to overweight negative rather than positive signals. The same is true for Ertac (2011), who uses a different signal structure, making her results difficult to compare directly. Kuhnen (2015) finds that subjects react more strongly to bad outcomes relative to good outcomes when these take place in a loss domain, but not when they take place in a gain domain. Thus, the degree to which people update asymmetrically is still very much an open question.

Resolving this question is important, because updating biases are a potential source of overconfidence, which is probably the most prominent and most discussed phenomenon in the literature on belief biases. Hundreds of studies have demonstrated that people are generally overconfident about their own ability and intelligence (see Moore and Healy 2008 for an overview). Overconfidence has been cited as a reason for over-entry into self-employment (Camerer and Lovallo 1999; Koellinger et al. 2007) as well as the source of suboptimal financial decision making (Barber and Odean 2001; Malmendier and Tate 2008). As a result, overconfidence is generally associated with both personal and social welfare costs.^1^

To see whether differences in updating do indeed explain economic decisions, we test whether they can predict the decision to enter a competition with another participant. Following Niederle and Vesterlund (2007), experimental studies which measure individual willingness to compete have received increasing attention. Their main finding is that, conditional on performance, women are less likely to choose a winner-takes-all competition over a non-competitive piece rate than men (see Croson and Gneezy 2009 and Niederle and Vesterlund 2011 for surveys, and Flory et al. 2015 for a field experiment). A growing literature confirms the external relevance of competition decisions made in the lab for predicting career choices. Buser et al. (2014) and Buser et al. (2017) show that competing in an experiment predicts the study choices of high-school students. Other studies have found correlations with the choice of entering a highly competitive university entrance exam in China (Zhang 2013), starting salary and industry choice of graduating MBA students (Reuben et al. 2015), as well as the investment choices of Tanzanian entrepreneurs (Berge et al. 2015) and monthly earnings in a diverse sample of the Dutch population (Buser et al. 2018).

Closest to our paper is an early version of MNNR, in which the authors construct individual measures of conservatism and asymmetry (Möbius et al. 2007). The authors conduct a follow-up competition experiment six weeks after the main experiment using a different task. They find that conservatism is negatively correlated with choosing the competition, while asymmetry is positively but insignificantly correlated. Our results go beyond this by changing the definition of the measures, so they are less likely to conflate asymmetry and conservatism. More importantly, our dataset is much larger. While Möbius et al. (2007) record four updating rounds per person for 102 individuals, we have data for 18 updating rounds over three different cognitive tasks for 297 individuals. This increases the precision of the individual measures and allows us to test whether individual updating tendencies are stable across tasks.

Finally, the results of our study are complementary to those of Ambuehl and Li (2018), who investigate subjects’ willingness to pay for signals of different informativeness and subsequent belief updating. In line with our findings, their results show that individual conservatism is consistent across a series of updating tasks that, unlike ours, are neutrally framed and have no ego-relevance. Conservatism also causes the willingness to pay for information to be unresponsive to increases in the signal strength, relative to a perfect Bayesian. Ambuehl and Li conjecture that conservatism may predict economic choices in less abstract environments. We confirm this conjecture by showing the relevance of updating biases for competitive behavior that has been shown to predict behavior outside the lab.

## Design

Our experimental design is based on MNNR. The experiment was programmed in z-Tree (Fischbacher 2007), and run at Aarhus University, Denmark, in the spring and summer of 2015. Overall, 22 sessions took place between April and September, with each session comprising between 8 and 24 subjects. Sessions lasted on average 70 minutes, including the preparation for payments. In total, 297 students from diverse study backgrounds participated in the experiment. Each session was composed of students with the same faculty, i.e. from either social science, science or the humanities.^2^ Students received a show-up fee of 40 Danish Crowns (DKK, $6.00 or €5.40).^3^ Average payment during the experiment was 176 DKK with a minimum of 20 and a maximum of 980 DKK.

Subjects read all instructions explaining the experiment on their computer screens. Additionally, they received a copy of the instructions in printed form.^4^ It was explained that the experiment would have four parts, one of which would be randomly selected for payment. Participants were told that the first three parts involved performance and feedback on a task as well as the elicitation of their beliefs, and that specific instructions for the last part would be displayed on the subjects’ screens after the first three parts were concluded. The instructions also specified that in each task each participant would be randomly matched with 7 others, and that their performance would be compared with the participants within that group.

We then explained the belief elicitation procedure. We elicited the probability about the event that participants were in the top half of their group of 8. To incentivize truthful reporting of beliefs, we used a variation of the Becker-DeGroot-Marshak(BDM) procedure, also known as “matching probabilities” or “reservation probabilities”. Participants were asked to indicate which probability *p* makes them indifferent between winning a monetary prize with probability *p*, and winning the same prize when an uncertain event *E* – in our experiment being in the top half – occurs. After participants indicate *p*, the computer draws a random probability and participants are awarded their preferred lottery for that probability. Under this mechanism, reporting the true subjective probability of *E* maximizes expected value, regardless of risk preferences (see Schlag et al. 2015for a more elaborate explanation, as well as a discussion of the origins of the mechanism). We explained this procedure, and stressed the fact that truthful reporting maximizes expected earnings, using several numerical examples to demonstrate this point. This stage took about 15 minutes including several control questions about the mechanics of the belief elicitation procedure.

Subjects then were introduced to the first of three different tasks. Each task was composed of a series of puzzles, and subjects were asked to complete as many puzzles as they could within a time frame of five minutes. Their score on the task would be the number of correct answers minus one-half times the number of incorrect answers. The first task, which we will refer to as “Raven”, consisted of a series of Raven matrices, where subjects have to select one out of eight options that logically completes a given pattern (subjects were told that “this exercise is designed to measure your general intelligence (IQ)”). In the second task, which we will refer to as “Anagram”, subjects were asked to formulate an anagram of a word displayed on the screen, before moving to the next word (subjects were told that “this exercise is designed to measure your ability for languages”). In the third task, which we will refer to as “Matrix”, subjects were shown a 3×3 matrix filled with numbers between 0 and 10, with two decimal places. The task was to select the two numbers that added up to 10 (subjects were told that “this exercise is designed to measure your mathematical ability”).^5^

The order of tasks was counterbalanced between sessions, in order to account for effects of depletion or boredom. The details for each task were explained only after the previous task had been completed. Subjects earned 8 DKK for each correct answer and lost 4 DKK for each incorrect answer. We explained to them that their payment could not fall below 0.

After each task, we elicited subjective beliefs about a subject’s relative performance. Specifically, we asked participants for their belief that they were in the top half of their group using the BDM procedure described above. After participants submitted their initial beliefs, we gave them a sequence of noisy but informative feedback signals about their performance. Participants were told that the computer would show them either a red or a black ball. The ball was drawn from one of two virtual urns, each containing 10 balls of different colors. If their performance was actually in the top half of their group, the ball would come from an urn with 7 black balls and 3 red balls. If their performance was not in the top half, the ball would come from an urn with 7 red balls and 3 black balls. Thus, a black ball constituted “good news” about their performance, a red ball “bad news”. After subjects observed the ball, they reported their belief about being in top half for a second time. This process was repeated five more times, resulting in six updating measurements for each participant for each task, and 18 belief updates overall. The prize at stake in the belief elicitation task was 10 DKK in each round of belief elicitation.

After the third task, subjects were informed about the rules of the fourth and final task, which consisted of the same kind of puzzles as the previous three tasks, mixed in equal proportions. Before performing this task, subjects were offered a choice between two payment systems, similar to Niederle and Vesterlund’s (2007) “Task 3”. The first option consisted of a piece-rate scheme, where the payment depended on their score in a linear way (12 DKK for a correct answer, -6 DKK for an incorrect one). The second option was to enter into a competition, where their score was compared to that of some randomly chosen other participant. If their score exceeded that of their matched partner, they would receive a payment of 24 DKK for each correct answer, and -12 DKK for each incorrect one. Otherwise, they would receive a payment of zero. In this round there was no belief elicitation.

After the competition choice, subjects were asked to fill out a (non-incentivized) questionnaire. Among other things, we asked how relevant participants thought the skills tested in each of the three tasks were for success in their field of study. We will use the answers to these questions as an individual measure of ego relevance of the tasks. Subjects also completed a narcissism scale, based on Konrath et al. (2014), and answered several questions related to their competitiveness, risk taking, and a range of activities which require confidence like playing sports or music on a high level.

## Do people update like Bayesians?

**Fig. 1:**
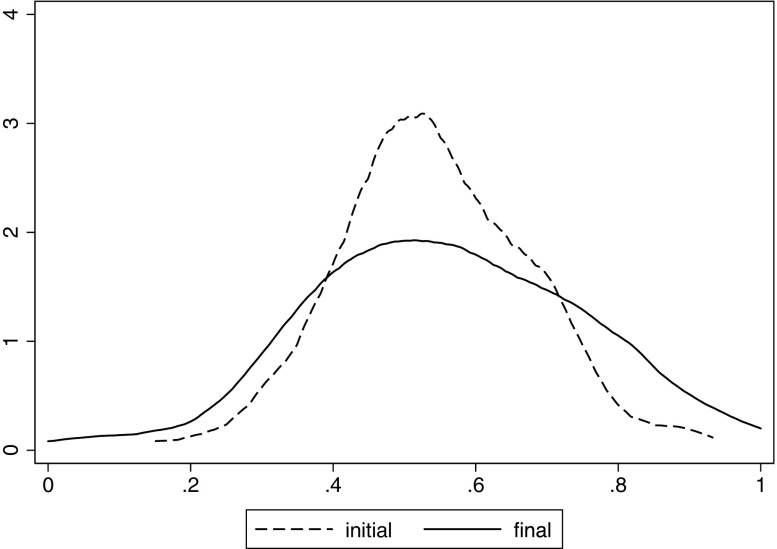
Density plots of initial and final belief distributions

In this section, we answer the question of whether people update in a Bayesian fashion, and focus on *aggregate* patterns of asymmetry and conservatism. To get a feeling for the aggregate distribution of beliefs, Fig. 1 shows the distributions of initial and final beliefs (that is, the beliefs the subjects held about being in the top half of their group before the first and after the sixth round of feedback, respectively) over all tasks. Mean initial beliefs are 54% (s.d. 0.13), indicating a modest amount of overconfidence, as only 50% can actually be in the top half. Average beliefs in the final round are roughly the same as in the initial round (55%), but the standard deviation increases to 0.19. This is likely to reflect an increase in accuracy, as the true outcome for each individual is a binary variable.

To understand whether beliefs have become better calibrated we run OLS regressions of initial and final beliefs in each task on the actual performance rank of subjects. In the initial round, we find that ranks explain more of the variation in beliefs in the Matrix task (*R*^2^ = 0.30) than in the Anagram task (*R*^2^ = 0.21) or the Raven task (*R*^2^ = 0.14). In each task, we find that the *R*^2^ of the model increases between 7 and 9 percentage points between the first and last round of belief elicitation. Thus, on average feedback does indeed succeed in providing a tighter fit between actual performance and beliefs over time.

### Updating mistakes

**Fig. 2:**
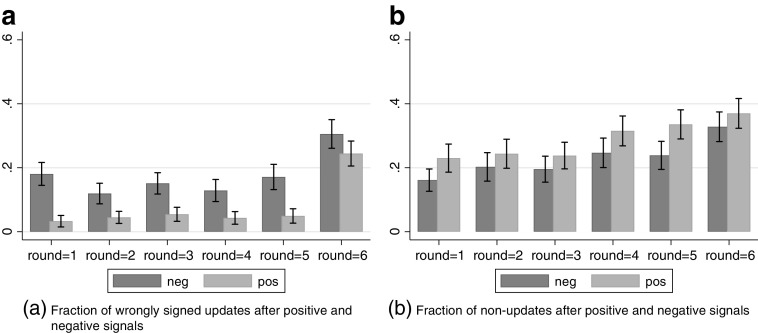
Overview of updating mistakes. The *x*-axis shows the feedback rounds, the *y*-axis shows the fraction of wrongly signed updates (left panel) or zero updates (right panel) after positive and negative feedback

We first look at one of the most basic requirements for updating, namely whether people change their beliefs in the right direction. Figure 2 shows the number of wrongly signed updates in each task. Per task and round, subjects update in the wrong direction in around 10% of the cases, when we average over positive and negative feedback. Interestingly, these updating mistakes display an asymmetric pattern, and the proportion of mistakes roughly doubles when the signal is negative. This result is highly significant in a regression of a binary indicator of having made an updating mistake on a dummy indicating that the signal was positive (*β* = − 0.077,*p* < 0.001).^6^ Thus, wrong updates are not pure noise, but seem to be partly driven by self-serving motives.

The right panel of Fig. 2 shows the fraction of another kind of updating mistake, namely the failure to update in any given round. The figure shows that on average about 25% of subjects do not update at all, a finding that is slightly lower than in Coutts (2018) and MNNR who find 42% and 36% respectively. In contrast to wrong updates, non-updating is more prevalent after receiving *positive* rather than negative feedback. Using the same test as for wrongly signed signals, we find that this difference is highly significant (*β* = 0.062,*p* < 0.001).

Thus, overall about one-third of our observations display qualitative deviations from Bayesian updating. Importantly, both zero and wrongly signed updates increase in the final updating rounds of each task. Whatever the reason for this pattern (perhaps subjects got bored, or they make more mistakes when they approach the boundaries of the probability scale),^7^ it implies that eliciting more than five updates on the same event is problematic. Gathering a substantial amount of data necessitates the introduction of several events – or tasks, as in the present study – about which to elicit probabilities (see also Barron 2016).

### Updating and prior beliefs

**Fig. 3:**
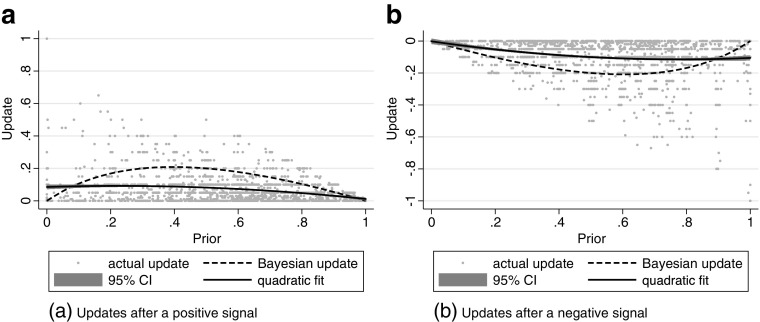
Overview of updating behavior. The *x*-axis shows prior beliefs, the *y*-axis shows the size of the update. The dashed line presents the Bayesian benchmark update. The solid line, with 95% confidence interval, presents the best quadratic fit to the data. We added a horizontal jitter to distinguish individual data points, updates in the wrong direction are excluded

We now focus on the relation between updates and prior beliefs. Figure 3 shows all combinations of the individual updates (*y*-axis) and prior beliefs (*x*-axis) presented as dots, excluding updates in the wrong direction. The dashed line presents the Bayesian or rational benchmark, showing that updates should be largest for intermediate priors that represent the largest degree of uncertainty. The solid line presents the best quadratic fit to the data, with a 95% confidence interval around it.

The left panel of Fig. 3 shows updating patterns after a positive signal. Two observations stand out. First, for all but the most extreme prior beliefs, updates are smaller than the Bayesian benchmark. This indicates that people are “conservative” on average. Second, the shape of the fitted function is flatter than that of the Bayesian benchmark. The right panel of Fig. 3 shows updates after a negative signal, and reveals very similar patterns in the negative domain.

It therefore appears that, in contrast to the Bayesian prescription, subjects on average update a constant absolute amount, without conditioning on prior beliefs.^8^ Alternatively, subjects with more extreme priors may somehow be better at Bayesian updating. To test this possibility, we re-estimated the quadratic fit in Fig. 1 with individual fixed effects, using only within subject variation in updates. We find a very similar result, showing that the failure to respond to the prior holds within subjects and is not due to differing updating capabilities between individuals.

### Regression analysis

To investigate asymmetry and conservatism more systematically, we follow MNNR in estimating the regression model of a linearized version of Bayes’ formula given by

1 $$ logit(\mu_{int}) = \delta logit(\mu_{in,t-1})+\beta_{H} 1_{(s_{int}=H)}\lambda_{H} +\beta_{L} 1_{(s_{int}=L)}\lambda_{L} +\epsilon_{int}.  $$

Here, *μ*_*int*_ represents the posterior belief for person *i* in task *n* ∈{Anagram, Raven, Matrix} after signal in round *t* ∈{1,2,3,4,5,6}, and *μ*_*in*,*t*− 1_ represents the prior belief (i.e. the posterior belief in the previous round). Thus, our belief data have a panel structure, with variation both across individuals and over rounds. *λ*_*H*_ is the natural log of the likelihood ratio of the signal, which in our case is 0.7/0.3 = 2.33, and *λ*_*H*_ = −*λ*_*L*_. $1_{(s_{int}=H)}$ and $1_{(s_{int}=L)}$ are indicator variables for a high and low signal respectively. The standard errors in all our regressions are clustered by individual.^9^

From the logistic transformation of Bayes’ rule one can derive that *δ*,*β*_*H*_,*β*_*L*_ = 1 correspond to perfect Bayesian updating (see MNNR for more details). Conservatism occurs if both *β*_*H*_ < 1 and *β*_*L*_ < 1, i.e. subjects place too little weight on either signal. If *β*_*H*_≠*β*_*L*_, this implies “asymmetry”, i.e. subjects place different weight on good signals compared to bad signals. MNNR find that *β*_*H*_ > *β*_*L*_ on an IQ quiz, but not on a neutral updating task.

The first column of Table 1 shows the results of all tasks pooled together, excluding all observations for a given task if the subject hits the boundaries of 0 or 1. In Column (2) we include only updates on tasks where a subject does not have any wrongly signed updates, and in Column (3) we restrict the data to the first four updating rounds in each task, to make the analysis identical to MNNR and avoid the noisy last two rounds.^10^

In each of the three columns, we see clear evidence for conservatism: both the coefficients on the positive and negative signal are very far from unity, the coefficient consistent with Bayesian updating. This implies that most subjects in our sample are indeed conservatively biased in their updating.^11^ The evidence for asymmetry is more mixed. The Wald test that both signals have the same coefficient, reported in the rows just below the coefficients, provides strong evidence for asymmetry in Column (1) only. In Columns (2) and (3) asymmetry is not statistically significant. Thus, it seems that asymmetry occurs only in wrongly signed updates, in line with Fig. 2. This evidence for asymmetry is weaker than that found in MNNR, who find a strong effect even when individuals making updating “mistakes” are excluded. The lack of a clear finding of robust asymmetry is in line with null-findings in several other studies cited above.^12^

**Table 1:** Regression results for model (1)

	(1)	(2)	(3)
Logit prior (*δ*)	0.860***	0.951***	0.948***
	(0.017)	(0.010)	(0.013)
Signal high (*β*_*H*_)	0.358***	0.404***	0.476***
	(0.018)	(0.022)	(0.020)
Signal low (*β*_*L*_)	0.254***	0.398***	0.464***
	(0.017)	(0.020)	(0.019)
*p* (Asymmetry)	0.000	0.759	0.583
No boundary priors in task	✓	✓	✓
No wrong updates in task		✓	✓
Only rounds 1-4			✓
Observations	4507	2197	2375
Subjects	288	218	272

All tasks are pooled. Columns reflect different sample selection criteria. Stars reflect significance in a test of the null hypotheses that coefficients are equal to 1 (not 0), *p* < 0.10, ** *p* < 0.05, *** *p* < 0.01

In the Appendix at the end of this paper, we reproduce some further graphical and statistical analysis to compare our results to MNNR’s. For instance, we investigate whether signals from preceding rounds matter for updating behavior. We find that lagged signals have a significant but small impact in our data. We also split our samples to investigate updating by gender, ego-relevance and IQ.

#### **Summary 1**

*We find that subjects deviate systematically from Bayesian updating:*

1. *about 10% of updates are in the wrong direction, and such mistakes are* *more likely after a negative signal,*
2. *one quarter of the updates are of size zero, and zero updates happen* *more often after a positive signal,*
3. *among the updates that go in the right direction, updates are a)* *not sufficiently sensitive to the prior belief, b) too conservative and c)* *symmetric with respect to positive and negative signals.*

## Measuring individual responsiveness to feedback

We now turn to the heterogeneity in updating behavior across subjects. In this section, we therefore define individual measures of asymmetry and conservatism. To quantify subjects’ deviations from others, we use the distance of each update from the average update by people with the same prior and the same signal. We call the resulting measures “relative asymmetry” (*RA*) and “relative conservatism” (*RC*), to reflect the nature of the interpersonal comparison. We use the absolute size of deviations, since using the relative size leads to large variations in our measures for individuals with extreme priors where average updates are small.^13^

To calculate individual deviations, we use residuals of the following regression model, which is run separately for positive and negative signals.

2 $$ {\Delta}\mu_{int}=\beta_{1}\mu_{in,t-1}+\beta_{2}\mu_{in,t-1}^{2}+\gamma_{1}1_{1}+\gamma_{2}1_{2}+...+\gamma_{10}1_{10}+\epsilon_{int}  $$

Here Δ*μ*_*int*_ := *μ*_*int*_ − *μ*_*in*,*t*− 1_ is the update by individual *i* in feedback round *t* and task *n* and 1_1_,1_2_...1_10_ represent dummies indicating that 0 ≤ *μ*_*in*,*t*− 1_ < 0.1,0.1 ≤ *μ*_*in*,*t*− 1_ < 0.2,...,0.9 ≤ *μ*_*in*,*t*− 1_ ≤ 1 respectively. These dummies introduce an additional (piecewise) flexibility to our predicted average updates compared to the quadratic fit shown in Fig. 3. The residuals of this regression thus measure individual deviations from the average update for either positive or negative signals, conditional on the prior of each individual.

For each individual *i* and for each round *t* and task *n*, regression residuals from Eq. 2 are denoted by *𝜖*_*int*_. Our measure of relative asymmetry in task *n* is then defined as

3 $$ RA_{in}:=\frac{1}{N_{in}^{-}}{\sum\limits}_{t = 1}^{6}1_{(s_{int}=L)}*\epsilon_{int}+ \frac{1}{N_{in}^{+}}{\sum}_{t = 1}^{6} 1_{(s_{int}=H)}*\epsilon_{int},  $$

where $N_{in}^{+}$ and $N_{in}^{-}$ are the observed number of positive and negative signals respectively. Thus, *RA*_*in*_ is the sum of the average residual after a positive and the average residual after a negative signal. It is positive if an individual updates a) upwards more than the average person after a positive signal, and/or b) downwards less than the average person after a negative signal.

To obtain an overall individual measure for relative asymmetry we calculate an analogous measure across all 3 tasks, spanning 18 updating decisions.

4 $$ RA_{i}:=\frac{1}{N_{i}^{-}}{\sum\limits}_{t = 1}^{18}1_{(s_{it}=L)}*\epsilon_{it}+ \frac{1}{N_{i}^{+}}{\sum\limits}_{t = 1}^{18} 1_{(s_{it}=H)}*\epsilon_{it},  $$

Correspondingly, relative conservatism for person *i* on task *n* is defined as

5 $$ RC_{in}:=\frac{1}{N_{in}^{-}}{\sum\limits}_{t = 1}^{6}1_{(s_{int}=L)}*\epsilon_{int}- \frac{1}{N_{in}^{+}}{\sum\limits}_{t = 1}^{6}1_{(s_{int}=H)}*\epsilon_{int}.  $$

In words, *RC*_*in*_ is the average residual after a negative signal minus the average residual after a positive update. Thus, *RC*_*in*_ is positive if an individual updates upward less than average after a positive signal and updates downward less than average after a negative signal. To obtain an overall individual measure of conservatism we calculate an analogous measure across all 3 tasks, spanning 18 updating decisions.

6 $$ RC_{i}:=\frac{1}{N_{i}^{-}}{\sum\limits}_{t = 1}^{18}1_{(s_{it}=L)}*\epsilon_{it}- \frac{1}{N_{i}^{+}}{\sum\limits}_{t = 1}^{18}1_{(s_{it}=H)}*\epsilon_{it}.  $$

These measures are similar to the ones developed by Möbius et al. (2007). One difference is that we use a more flexible function to approximate average updating behavior. A second, more important difference is that we give equal weight to positive and negative updates which avoids conflating asymmetry and conservatism for subjects with an unequal number of positive and negative signals. For example, a subject who is relatively conservative and receives more positive than negative signals would have a negative bias in asymmetry, as the downward residuals after a positive signal would be overweighted relative to the upward residuals after a negative signal.

Finally, updates in the wrong direction pose a problem for the computation of our relative measurements. An update of the wrong sign has a potentially large impact on our measures, as it is likely to result in a large residual. However, as it seems likely that such updates at least partly reflect “mistakes”, this may unduly influence our measures. To mitigate this effect, we treat wrongly signed updates as zero updates in the calculation of our individual measures. Note also that we only calculate our measures for subjects who receive at least one positive and at least one negative signal as it is impossible to distinguish *RC* from *RA* for those with only positive or only negative signals.

## Consistency and impact of feedback responsiveness

We now analyze these measures of responsiveness, looking in turn at their consistency across tasks, their variation across ego-relevance and their impact on post-feedback beliefs.

### Consistency of feedback responsiveness across tasks

An important motivating question for our research is whether feedback responsiveness can be considered a trait of the individual. To answer this question, we look at the consistency of *RC* and *RA* across tasks. Table 2 displays pairwise correlations between our measures over tasks. For *RC*, we find highly significant correlations in the range of 0.22–0.37. For *RA*, correlations are smaller, and the only significant correlation is that between *RA* in the Matrix and Anagram tasks. This latter result is puzzling, as these tasks are very different from each other and are seen as relevant by different people.^14^

**Table 2:** Spearman’s pairwise correlations of measures over task

	RC(M)	RC(R)		RA(M)	RA(R)
RC(A)	0.218***	0.365***	RA(A)	0.149**	− 0.043
RC(M)		0.234***	RA(M)		0.099

* *p* < 0.10, ** *p* < 0.05, *** *p* < 0.01. A stands for “Anagram”, M stands for “Matrices” and R stands for “Raven”

#### **Summary 2**

*For a given individual, relative conservatism displays robust correlation over* *tasks, whereas relative asymmetry does not.*

### Ego-relevance and gender effects

We now turn to an analysis of heterogeneity in feedback responsiveness related to task and subject characteristics. In turn, we discuss the role of ego-relevance and gender.

#### The effect of ego-relevance

Past research suggests that the ego-relevance of a task changes belief updating, and can trigger or increase asymmetry and conservatism (see Section 2), indicating that responsiveness to feedback is motivated by the goal of protecting a person’s self-image. Furthermore, Grossman and Owens (2012) show that ego-relevance also leads to initial overconfidence in the form of higher priors.

The variation in study background in our experimental sample allows us to study this directly, as it creates variation in the ego-relevance of the different experimental tasks. We measured the relevance of each task with a questionnaire item.^15^ We conjecture that participants who attach higher relevance to a particular task will be more confident and will update more asymmetrically. Furthermore, if subjects are more confident in tasks that they consider more relevant, they would have an ego motivation to be more conservative as well in order to protect any ego utility they derive from such confidence. To see whether these conjectures are borne out in the data, we first investigate whether relevance affected subjects’ beliefs about their own relative performance before they received any feedback. To this end, we regress initial beliefs in each task on the questionnaire measure of relevance. We also include a gender dummy in these regressions, which is discussed below.

The results, reported in Table 3, show that relevance has a highly significant effect on initial beliefs. Heterogeneity in scores can only explain part of this effect, as we show in Column (2) where we control for scores and performance ranks within the session. The last two columns show that the effect of relevance on initial beliefs is robust to the introduction of individual fixed effects. This implies that the effect stems from within-subject variation in relevance across tasks. That is, the same individual is more confident in tasks that measure skills that are more ego-relevant.

**Table 3:** OLS regressions of initial beliefs on task relevance and gender

	(1)	(2)	(3)	(4)
Female	− 0.050***	− 0.030**		
	(0.015)	(0.014)		
Relevance	0.029***	0.021***	0.031***	0.023***
	(0.004)	(0.004)	(0.005)	(0.004)
Scores & ranks		✓		✓
Individual fixed effects			✓	✓
N	891	891	891	891

Fixed effects regressions with the same outcome variable are reported in the same column. * *p* < 0.10, ** *p* < 0.05, *** *p* < 0.01. Standard errors are clustered at the individual level

This result is consistent with the idea that confidence is ego-motivated: participants who think a task is more relevant to the kind of intelligence they need for their chosen career path are more likely to rate themselves above others. Alternatively, it could mean that people choose the kind of studies for which they hold high beliefs about possessing the relevant skills. Note however that the pattern cannot be explained by participants who think that their study background gives them an advantage over others, as they knew that all other participants in their session had the same study background.

To test the extent to which ego-relevance can explain the variation in feedback responsiveness across tasks, we regress *RA* and *RC* for each task on the relevance that an individual attaches to that task. We again control for gender in the regressions. The results in Table 4 show that the impact of relevance on both *RA* and *RC* is positive. For *RA*, the estimated coefficient is small and insignificant. For *RC*, the effect is statistically significant and, moreover, robust to controlling for scores, ranks and initial beliefs. In the regressions reported in the lower part of the table (Columns 1a-6a), we add individual fixed effects to compare more and less relevant tasks within subject, disregarding between-subject variation. The effect is equally strong, indicating that the same subject is more conservative in tasks that measure skills which are more ego-relevant.

**Table 4:** OLS regressions of asymmetry (*RA*) and conservatism (*RC*) on task relevance and gender

	(1)	(2)	(3)	(4)	(5)	(6)
	RA	RC	RA	RC	RA	RC
Female	− 0.108	0.183**	− 0.060	0.179**	− 0.048	0.181**
	(0.077)	(0.085)	(0.073)	(0.084)	(0.073)	(0.084)
Relevance	0.023	0.040*	0.009	0.041*	0.002	0.039*
	(0.022)	(0.022)	(0.021)	(0.022)	(0.021)	(0.022)
Scores & ranks			✓	✓	✓	✓
Initial beliefs					✓	✓
	(1a)	(2a)	(3a)	(4a)	(5a)	(6a)
Relevance	0.026	0.039*	0.017	0.036	0.021	0.039*
	(0.028)	(0.022)	(0.026)	(0.023)	(0.026)	(0.023)
Scores & ranks			✓	✓	✓	✓
Initial beliefs					✓	✓
Individual fixed effects	✓	✓	✓	✓	✓	✓
N	798	798	798	798	798	798

* *p* < 0.10, ** *p* < 0.05, *** *p* < 0.01. Standard errors are clustered at the individual level. Each person-task combination is one observation. Regressions with asymmetry as the outcome additionally control for conservatism and vice versa

Combined with the positive effect of relevance on initial beliefs, the results are consistent with the idea that people deceive themselves into thinking that they are good at ego-relevant tasks and become less responsive to feedback in order to preserve these optimistic beliefs.

#### **Summary 3**

*We find that the self-reported ego relevance of the task is positively correlated* *with initial beliefs and relative conservatism. We do not find a correlation* *between ego relevance and relative asymmetry.*

#### Gender effects

Earlier studies have consistently found that women are less (over)confident than men, especially in tasks that are perceived to be more masculine (see Barber and Odean 2001 for an overview). In line with this literature, Table 3 shows that women are about 3 percentage points less confident about being in the top half of performers across all three tasks, after controlling for ability.

MNNR, Albrecht et al. (2013) and Coutts (2018) find that women also update more conservatively. We replicate this result using our individual measure in Table 4, where we see a significant negative effect of a female dummy on individual conservatism across the three tasks, an effect that is robust to controlling for scores and initial beliefs. We do not find a significant gender difference in *RA*.

#### **Summary 4**

*Women are initially less confident and update more conservatively than* *men.*

### Impact of feedback responsiveness on final beliefs

To understand the quantitative importance of heterogeneity in feedback responsiveness, we look at the effect on the beliefs in the final round of each task. As the impact of relative conservatism and asymmetry depends on received feedback, we run a linear regression of the form

7 $$\begin{array}{@{}rcl@{}} \mu_{in}&=&{\beta^{C}_{0}} * RC_{in} + {\beta^{A}_{0}} * RA_{in} + {\sum\limits}_{s = 1}^{5} \beta_{s} 1_{(s^{+}_{in}=s)} + {\sum\limits}_{s = 1}^{5} {\beta^{C}_{s}} 1_{(s^{+}_{in}=s)}*RC_{in}\\ &&+{\sum\limits}_{s = 1}^{5} {\beta^{A}_{s}} 1_{(s^{+}_{in}=s)}*RA_{in} +\varepsilon_{in}, \end{array} $$

where *μ*_*in*_ is the final belief after the last round of feedback, and $1_{(s^{+}_{in}= 1)},1_{(s^{+}_{in}= 2)},...,1_{(s^{+}_{in}= 5)}$ represent dummies taking a value of 1 if subject *i* got the corresponding number of positive signals in task *n*. *RA*_*in*_ and *RC*_*in*_ are defined as in Eqs. 3 and 5 above.

**Fig. 4:**
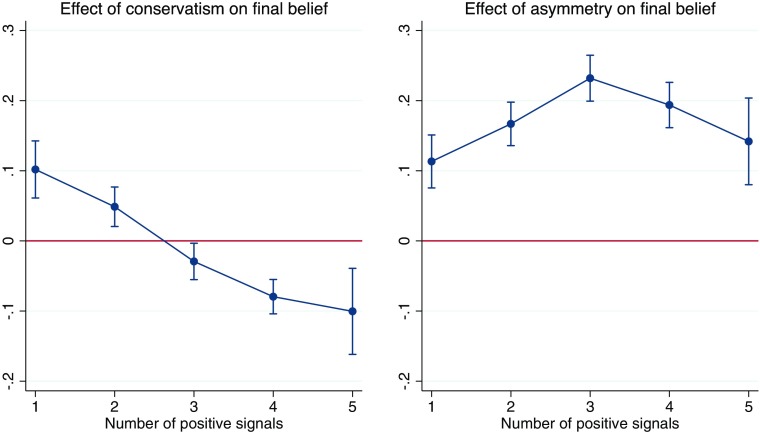
The impact of an increase of one standard deviation in *RA*/*RC* on final beliefs after the last updating round, split by the number of positive signals

The left panel of Fig. 4 shows the effect of an increase of one standard deviation in conservatism, separately for each number of *s* positive signals received, i.e. ${\beta ^{C}_{0}}+{\beta ^{C}_{s}} $. The data confirm that conservatism raises final beliefs for people who receive many bad signals and lowers them for people who receive many good signals, cushioning the impact of new information. The right panel of Fig. 4 shows a similar graph for the effect of a standard deviation increase in asymmetry, i.e. ${\beta ^{A}_{0}}+{\beta ^{A}_{s}} $. The impact of asymmetry is to raise final beliefs for any combination of signals. The effect is highest when signals are mixed, as the absolute size of the belief updates, and hence the effect of asymmetry, tend to be larger in this case.

The direction of the effects shown in Fig. 4 is implied by our definitions, and should not be surprising. More interesting is the size of the effects of both *RA* and *RC*. For subjects with unbalanced signals, conservatism will matter more. Specifically, an increase of one standard deviation in *RC* raises final beliefs by 10 percentage points for subjects who received 1 positive and 5 negative signals, and lowers them by about the same amount for people who receive 5 positive and 1 negative signals. By contrast, asymmetry is most important for people who saw a more balanced pattern of signals. A one standard deviation increase in *RA* leads to an average increase in post-feedback beliefs of over 20 percentage points for a person who saw 3 good and 3 bad signals, and therefore should not have adjusted beliefs at all.

In each individual task, the standard deviation of final beliefs is about 30 percentage points, implying that for any realization of signals, variation in feedback responsiveness explains a substantial part of the variation in final beliefs. In fact, the adjusted *R*^2^ of our regression model in Eq. 7 is 67%, which falls to 46% when we drop the responsiveness measures and their interactions from the model. Thus our responsiveness measures explain about an additional 21 percentage points of total variation in final beliefs after controlling for signals.

To investigate whether feedback measures explain beliefs within subjects across tasks or between subjects, we run the same regression including individual fixed effects. The within-subjects (across-tasks) *R*^2^ is 0.68 with and 0.50 without our responsiveness measures, while the between-subjects *R*^2^ is 0.65 with and 0.43 without our responsiveness measures. This demonstrates that there is meaningful individual heterogeneity in responsiveness to feedback and that relative conservatism and asymmetry are important determinants of individual differences in belief updating and confidence.

#### **Summary 5**

*When observing unbalanced feedback with many more positive than negative* *signals or vice versa, a one standard deviation change in relative conservatism* *or asymmetry changes final beliefs by a little over 10 percentage points. For* *balanced feedback with similar amounts of both positive and negative signals,* *a one standard deviation change in asymmetry changes final beliefs by about* *20 percentage points. Controlling for feedback content, relative conservatism* *and asymmetry jointly explain an additional 21 percentage points of the* *between-subjects variation in final beliefs.*

## Predictive power of feedback responsiveness

In this section we investigate the predictive power of feedback responsiveness for the choice to enter a competition. Competition was based on the score in the final task of the experiment, which consisted of a mixture of Matrix, Anagram and Raven exercises. Before they performed this final task, subjects decided between an individual piece-rate payment and entering a competition with another subject, as described in Section 3.

The posterior beliefs about the performance in the previous tasks are likely to influence this decision, which implies that our measures of feedback responsiveness should matter. Specifically, we would expect that relative asymmetry raises the likelihood of entering a competition because it inflates self-confidence. The hypothesized effect of relative conservatism is more complex. Conservatism raises final beliefs, and supposedly competition entry, for those who have many negative signals. However, it should depress competition entry for those who received many positive signals. In addition to this belief channel, it may be that updating behavior is correlated with unobserved personality traits that affect willingness to compete.

To investigate these hypotheses, we run probit regressions of the (binary) entry decision on *RA* and *RC*, controlling for ability. We also include gender, as it has been shown that women are less likely to enter a competition (Niederle and Vesterlund 2007), a finding we confirm in our regressions. The results are reported in Table 5. Column (1) controls for ability (assessed by achieved scores and performance ranks), but not beliefs, and shows that both conservatism and asymmetry have a positive effect on entry. The coefficient for asymmetry is not affected when we control for the number of positive signals or initial beliefs (Columns 2-3), but virtually disappears when we control for final beliefs (Columns 4-5). This shows that asymmetry affects entry only through its effect on final beliefs rather than through a correlation with any unobserved characteristics.

**Table 5:** Probit regressions of competition entry on standardized measures of feedback responsiveness

	(1)	(2)	(3)	(4)	(5)
Female	− 0.121**	− 0.113**	− 0.070	− 0.088*	− 0.082*
	(0.048)	(0.048)	(0.046)	(0.045)	(0.046)
Rel. Asymmetry (*RA*)	0.079***	0.105***	0.066***	0.023	0.044
	(0.025)	(0.029)	(0.024)	(0.025)	(0.032)
Rel. Conservatism (*RC*)	0.053**	0.221***	0.045*	0.048**	0.134*
	(0.024)	(0.076)	(0.023)	(0.022)	(0.075)
Rel. Conservatism x # pos. signals		− 0.018**			− 0.009
		(0.008)			(0.008)
# pos. signals		0.024*			0.006
		(0.013)			(0.013)
Scores and ranks	✓	✓	✓	✓	✓
Initial beliefs			✓	✓	✓
Final beliefs				✓	✓
N	297	297	297	297	297

Marginal effects reported, robust standard deviations in parentheses. * *p* < 0.10, ** *p* < 0.05, *** *p* < 0.01

In order to better understand the effect of conservatism, we interact *RC* with the amount of positive signals. The estimated coefficients show that for a person with no positive signals, an increase of one standard deviation in *RC* raises the probability of entry by 22 percentage points, an effect that is larger than the gender effect. If we compare the coefficient of the interaction term with the coefficient of the number of positive signals in Column (2), we see that an increase in *RC* reduces the effect of a positive signal by about 75% (0.018/0.024). The estimated total effect of *RC* is negative for someone with large amounts of positive signals. The effect of more positive signals and its interaction with *RC* disappear when we control for initial and final beliefs (Column 5), confirming that these effects indeed go through beliefs. However, *RC* still exerts a large positive direct effect. Together, Columns (4) and (5) clearly suggest that, in addition to its effect on final beliefs, there is an effect of *RC* that may be a part of a person’s personality. Controlling for the relevance that subjects attach to the three tasks does not alter any of the results.^16^

### **Summary 6**

*Relative asymmetry raises the probability of competition entry by increasing* *final beliefs. Relative conservatism raises the probability of competition entry* *for people with many negative signals, and diminishes it for those with many* *positive signals. Conservatism also has an independent, positive effect on entry,* *suggesting it may be correlated with competitive aspects of personality.*

## Discussion and conclusion

This paper contains a comprehensive investigation of Bayesian updating about beliefs in own ability. We investigate both aggregate patterns of asymmetry and conservatism and individual heterogeneity in these dimensions. On aggregate, we find strong evidence for conservatism and little evidence for asymmetry. Our individual measures of relative feedback responsiveness deliver a number of new insights about individual heterogeneity. We find that differences in relative conservatism are correlated across tasks that measure different cognitive skills, indicating that they can be considered a characteristic or trait of the individual. The same cannot be said about relative asymmetry, which is not systematically correlated across tasks. We also find that individuals are more conservative, but not more asymmetric, in tasks that they see as more ego-relevant. Both measures have substantial explanatory power for post-feedback confidence and competition entry. Relative conservatism affects entry both through beliefs and independently, whereas relative asymmetry increases entry by biasing beliefs upward. Finally, we find that women are significantly more conservative than men.

Our study demonstrates both the strengths and limitations of our measurements of asymmetry and conservatism. Measuring updating biases is complex. There is noise in our measures, and their elicitation is relatively time consuming. Future research could investigate whether simpler or alternative measures could deliver similar or better predictive power. Another approach would be to vary the belief elicitation mechanism (Schlag et al. 2015). Since subjects do not appear to be particularly good at Bayesian updating, it would also be interesting to look at the results through the lens of alternative theoretical models. For instance, some models allow for ambiguity in prior beliefs, and may provide a richer description of beliefs about own ability (see Gilboa and Schmeidler 1993).

Nevertheless, our results hold promise for researchers in organizational psychology and managerial economics, where feedback plays a central role. Specifically, an interesting research area would be to investigate the predictive power of these measures in the field. It would be interesting to correlate relative conservatism and asymmetry with decisions such as study choice or the decision to start a (successful) business, as well as a range of risky behaviors in which confidence plays a central role. In doing so, it could follow research that has linked laboratory or survey measurements of personal traits to behavior outside the lab. For instance Ashraf et al. (2006), Meier and Sprenger (2010), Almlund et al. (2011), Moffitt et al. (2011), Castillo et al. (2011), Sutter et al. (2013) and Golsteyn et al. (2014) link self-control, patience, conscientiousness and risk attitudes to outcomes in various domains such as savings, education, occupational and financial success, criminal activity and health outcomes. If such a research program were successful, it could reduce the costs of overconfidence and underconfidence to the individual and to society as a whole.

### Electronic supplementary material

Below is the link to the electronic supplementary material.

(PDF 837 KB)

## Figures and Tables

**Fig. 5:**
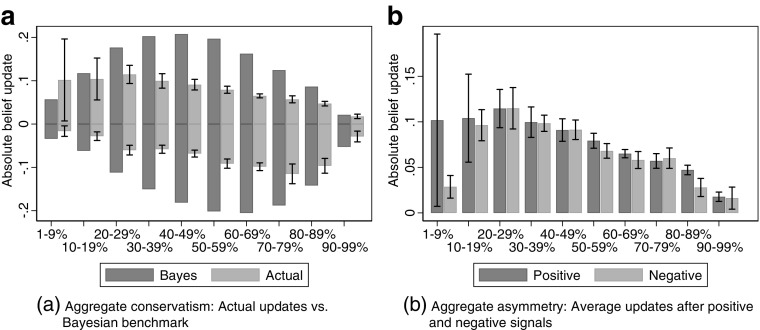
Overview of updating behavior, reproducing graphs in MNNR. The *x*-axis shows categories of prior beliefs, the *y*-axis shows the average size of the updates. 95% confidence intervals are included. Updating data come from round 1-4, updates in the wrong direction are excluded, replicating exactly the sample selection rules of MNNR

## Appendix: Comparison to MNNR

In this Appendix we reproduce some of the analysis in MNNR with our data, using the same sample selection criteria.^17^ Figure 5 reproduces the main graphs in MNNR with our data. Panel (a) shows the actual updates after both a positive and a negative signal as a function of prior belief, indicating the rational Bayesian update in dark bars as a benchmark. It is immediately clear that updating is conservative, as subjects update too little in both directions. To investigate asymmetry, panel (b) of Fig. 5 puts the updates in the two directions next to each other. In contrast to the results by MNNR, no clear pattern emerges. While updates after a negative update are slightly smaller for some priors this difference is not consistent.

As outlined in Section 4, MNNR also use a logistical regression framework to statistically compare subjects’ behavior to the Bayesian benchmark. To supplement our main replication in the main text, we provide here additional results conditioning on ego-relevance of the task, gender and IQ. Table 6 reports the results of regressions based on various sample splits. In Columns (1) and (2), we estimate the response to positive and negative signals separately for observations with above or below-median task relevance. The post-estimation Wald tests reported below the coefficients reveal no significant difference in aggregate conservatism or asymmetry when we compare the results by relevance. One reason for this could be that the (necessary) exclusion of observations from individuals who hit the boundaries likely excludes the least conservative (and most asymmetric) individuals.^18^

**Table 6:** Regression results for model (1), using various sample splits

		(1)	(2)		(3)	(4)		(5)	(6)
	Relevance low	0.849***	0.939***	Raven low	0.810***	0.908***	Men	0.914***	0.967*
Logit prior (*δ*)		(0.020)	(0.018)		(0.030)	(0.025)		(0.018)	(0.018)
	Relevance high	0.871***	0.960***	Raven high	0.919***	0.987	Women	0.805***	0.924***
		(0.025)	(0.015)		(0.025)	(0.022)		(0.027)	(0.018)
	Relevance low	0.359***	0.482***	Raven low	0.352***	0.494***	Men	0.405***	0.535***
Signal High (*β*_*H*_)		(0.022)	(0.024)		(0.027)	(0.034)		(0.027)	(0.031)
	Relevance high	0.353***	0.463***	Raven high	0.365***	0.474***	Women	0.316***	0.422***
		(0.027)	(0.027)		(0.029)	(0.033)		(0.022)	(0.024)
	Relevance low	0.276***	0.472***	Raven low	0.254***	0.489***	Men	0.303***	0.521***
Signal Low (*β*_*L*_)		(0.022)	(0.024)		(0.027)	(0.032)		(0.025)	(0.030)
	Relevance high	0.225***	0.457***	Raven high	0.301***	0.478***	Women	0.227***	0.415***
		(0.024)	(0.026)		(0.027)	(0.033)		(0.022)	(0.023)
Asymmetry	P(Asymmetry)	0.000	0.859	P (Asymmetry)	0.097	0.918	P (Asymmetry)	0.002	0.765
	(high)			(high)			(Men)		
	P(Asymmetry)	0.003	0.718	P(Asymmetry)	0.010	0.892	P(Asymmetry)	0.003	0.719
	(low)			(low)			(Women)		
Group differences	P(Prior, high	0.432	0.341	P(Prior, high	0.006	0.017	P(Prior, men	0.001	0.083
	vs low)			vs low)			vs women)		
	P(Conservatism,	0.235	0.522	P(Conservatism,	0.299	0.684	P(Conservatism,	0.002	0.001
	high vs low)			high vs low)			men vs women)		
	P(Asymmetry high	0.304	0.916	P(Asymmetry	0.527	0.866	P(Asymmetry,	0.767	0.877
	vs low)			high vs low)			men vs women)		
No boundary priors in task		✓	✓		✓	✓		✓	✓
No wrong updates in task			✓			✓			✓
Only rounds 1-4			✓			✓			✓
Observations		4507	2375		3020	1585		4507	2375
Subjects		288	272		281	250		288	272

All tasks are pooled, except in Column (3)-(4) where the Raven task is excluded. Columns (1) - (2) show sample split by median task relevance. Columns (3)-(4) show results of sample split by median IQ, as measured on the Raven test. Columns (5)-(6) show results of sample split by gender. Stars reflect significance in a test of the null hypotheses that coefficients are equal to 1 (not 0), *p* < 0.10, ** *p* < 0.05, *** *p* < 0.01

In Columns (3) and (4), we check whether a higher IQ translates into smaller updating biases. To do, so we split the sample by high and low IQ, as measured by a median split on the Raven test (“Raven low” vs. “Raven high”). To avoid endogeneity, we exclude the updates in the Raven test itself. We find that people who score low on the Raven test put lower weight on the prior. *δ* < 1 implies that beliefs will be biased towards 50% and this bias is stronger for low IQ subjects. In Columns (5) and (6), we estimate the response to positive and negative signals separately by gender. We find that women put less weight on the prior, compared to both Bayesians and men. In addition, we find the same significant gender difference in conservatism which we discovered using our individual measures.

Finally, we use the logistical regressions specification in the main text to investigate some further implication of Bayesian updating. Specifically, updates should only depend on the prior belief and the last signal, not on past signals, a property that MNNR call “sufficiency”. If sufficiency is violated, then any measure of individual feedback responsiveness (including ours) will necessarily depend on the order of signals. MNNR test for this by including lagged signals in their regression, and find that these are not significant, thus confirming sufficiency. In Table 7 we reproduce this exercise. We find that coefficients on past signals are positive and statistically significant. However, their impact on posteriors is smaller, by about a factor 8, than that of the last signal received. Thus, the sequence of signals has at most a modest impact on the individual measurements in our data.

**Table 7:** Results for regression model (1), with the additional inclusion of lagged signals

	(1)	(2)	(3)
	Round 1	Round 2	Round 3
Logit prior (*δ*)	0.924***	0.947***	0.943***
	(0.026)	(0.021)	(0.027)
Signal High (*β*_*H*_)	0.497***	0.419***	0.404***
	(0.033)	(0.026)	(0.037)
Signal Low (*β*_*L*_)	0.456***	0.423***	0.384***
	(0.030)	(0.028)	(0.031)
Signal (t-1)	0.059***	0.076***	0.073***
	(0.023)	(0.020)	(0.026)
Signal (t-2)		0.068***	0.066***
		(0.018)	(0.024)
Signal (t-3)			− 0.001
			(0.026)
No boundary priors in task	✓	✓	✓
No wrong updates in task	✓	✓	✓
Observations	600	600	575
Subjects	272	272	267

Lagged updates after a negative signal are multiplied by minus one. * *p* < 0.10, ** *p* < 0.05, *** *p* < 0.01

## Footnotes

1 Daniel Kahneman argues that overconfidence is the bias he would eliminate first if he had a magic wand. See http://www.theguardian.com/books/2015/jul/18/daniel-kahneman-books-interview.

2 In total, 101 social science students took part, of whom 51 were male, including students in Economics and Business Administration, International Business, Public Policy, Innovation Management, Economics and Management, Sustainability, and Law. In total 97 science students participated, of whom 55 were male, including students from Physics, Health Technology, Mathematics, Engineering, Geology, (Molecular) Biology, IT Engineering and Chemistry. Finally, 99 students from the humanities took part, among whom 30 were male, from study backgrounds like European Studies, International Relations, Human Security, Journalism, Japan Studies, Languages (mainly English, Spanish, Italian, German), Culture and Linguistics.

3 At the time of the experiment, the exchange rate of 1 DKK was $0.15 or €0.135.

4 All instructions and screen shots of the experimental program can be found in the Electronic Supplemental Materials.

5 We also implemented two different levels of difficulty for each task. The aim was to generate an additional source of diversity in confidence levels that could be used in our estimation procedures. As it turned out, task difficulty did not significantly affect initial confidence levels, so we pool the data from all levels of difficulty in our analysis. During the sessions subjects were always compared to other subjects who performed the task at the same difficulty level.

6 This result comes from OLS regressions with standard errors clustered on the individual level and individual fixed effects. The fraction of wrong updates in Fig. 2 is about the same as that found in MNNR, and four percentage points higher than in Schwardmann and van der Weele (2016), studies that use comparable belief elicitation mechanisms and a similar feedback structure. In the instructions, Schwardmann and van der Weele (2016) use a choice list with automatic implementation of a multiple switching point for the BDM design, rather than the slightly more abstract method of eliciting a reservation probability. This may explain the lower rate of wrong updates. Schwardmann and van der Weele (2016) find the same asymmetry in wrong updates when it comes to positive and negative signals. Charness et al. (2011) find more updating errors on ego-related tasks than on neutral tasks, but do not find asymmetry in these errors.

7 After multiple signals in the same direction, subjects will hit the “boundaries” of the probability scales. In our setting, about 10% of subjects declared complete certainty after the 5th round of signals.

8 Note that the decline in the absolute level of updates for high priors (left panel of Fig. 3) and low priors (right panel) is superficially in line with Bayes’ rule, but is in fact due to the boundary of the probability space, i.e. the possibility for upward (downward) updates narrows when the prior approaches one (zero). The linear decline in the maximum updates when approaching the boundaries of the updating space shows that this ceiling is indeed binding for a part of the subjects.

9 The design of MNNR includes four updating rounds and they omit all observations from subjects who ever update in the wrong direction or hit the boundaries. To make our results comparable, we exclude all observations for a given task for subjects who update at least once in the wrong direction or hit the boundaries of the probability space before the last belief elicitation, and also show results based on the first four rounds only.

10 In Columns (2) and (3) we exclude all data for an individual in a given task when there is a single update in the wrong direction. Since there was a strong increase in wrong updates in the last two rounds, excluding those rounds actually leads to an increase in the number of observations in the specification in Column (3).

11 These results are relevant to the discussion on “base-rate neglect”, the notion that subjects ignore priors or base-rates, and place too much weight on new information (Kahneman and Tversky 1973; Bar-Hillel 1980; Barbey and Sloman 2007). Like in base-rate neglect, our subjects are also insensitive to the size of the prior. However, they do not place *enough* weight on new information. An interesting question is why conservatism rather than base-rate neglect occurs in these tasks. One potential explanation is that conservatism may depend on the signal strength. Ambuehl and Li (2018) provide evidence for this hypothesis, and show that subjects are more conservative for more informative signals. They also find that the relative differences in update size between individuals remain rather stable across different signal structures, which implies that our individual measures of relative feedback responsiveness defined in Section 5 should be robust to changes in the signal strength.

12 Ertac (2011), Coutts (2018) and Schwardmann and van der Weele (2016) even find a tendency in the opposite direction. Ertac (2011) uses a different signal and event space, making it harder to compare her results to ours or MNNR’s.

13 We do not use deviations from the Bayesian benchmark for our personal measures. Doing so would lead these measures to reflect the impact of biases that are shared by all subjects, rather than meaningful differences between subjects. For instance, Fig. 3 shows that subjects with more extreme beliefs will *appear* closer to the Bayesian benchmark. However, as we showed in the previous section, this merely reflects differences in priors, not interpersonal differences in responsiveness to feedback.

14 As we show below, individuals who attach more relevance to a task tend to be more conservative in updating their beliefs about their performance in that task. However, the estimated correlations of conservatism across tasks are not due to correlations of relevance across tasks. If we regress individual conservatism in each task on individual relevance and correlate the residuals of this regression across tasks, the estimated correlations are very similar to the ones reported in Table 2.

15 We measured relevance in the final questionnaire. For instance, for the Raven task we asked the following question: “I think the pattern completion problems I solved in this experiment are indicative of the kind of intelligence needed in my field of study.” Answers were provided on a Likert scale from 1 to 7. Each subject answered this question three times, one time for each task. In a regression of relevance on study background, we do indeed find that students from a science background find the Raven task significantly more relevant than students from social sciences or humanities. Conversely, students from the humanities attach significantly more relevance to the Anagrams task and less to the Matrix task than either of the other groups. We also included a control for gender in these regressions, to account for the fact that our study background samples are not gender balanced.

16 Contrary to our results, Möbius et al. (2007) find that relative conservatism is negatively correlated with competition entry. Like us, they find that relative asymmetry positively predicts competition entry. However, it is difficult to compare their results to ours. Their measures are based on only 102 individuals and four updating rounds (versus 18 in our case) and their competition experiment is based on a task which is quite different from the one used in the main experiment. They enter conservatism and asymmetry in separate regressions which ignores the fact that for subjects with an unequal number of positive and negative signals, their measures of conservatism and asymmetry are mechanically correlated and one therefore picks up the effect of the other.
