# Supplementary material for: Responsiveness to feedback as a personal trait
Source: J Risk Uncertain. 2018 May 10;56(2):165–92. doi: 10.1007/s11166-018-9277-3 (PMC6445505; doi:10.1007/s11166-018-9277-3)
Supplement: Supplementary file 1 — (PDF 837 KB) [file 11166_2018_9277_MOESM1_ESM.pdf]

Electronic Supplemental Materials for "Responsiveness to Feedback as a Personal Trait"

by Thomas Buser, Leonie Gerhards and Joël van der Weele.
Journal of Risk and Uncertainty, 2018

This document provides the onscreen instructions for the experiment.

Welcome to this experiment.

Welcome to this experiment! Please refrain from communication for the duration of the experiment and make sure your cell-phone is switched off. Read and follow the instructions carefully; they contain everything you need to know to participate.

For showing up on time you receive 40.00 DKK. During the experiment you can earn additional money for yourself, depending on the decisions that you and other participants will make during the experiment. Your earnings will later be transferred to your NemKonto.

The experiment consists of four rounds. We will first explain you the first three rounds of the experiment, and give you the instructions for the fourth round of the experiment after you have finished the first three rounds. If you have questions at any point, raise your hand and the experimenter will come to your seat to answer them in private.

Note that all the other participants in this session are students from the same faculty as you.

Next

In each of rounds 1 to 3 of the experiment you will work on a different exercise. Each exercise consists of a number of puzzles. The kind of puzzle differs between the different exercises. Each exercise lasts 5 minutes and you will earn money for the number of correctly solved puzzles within this time limit.

After each exercise, we will ask you about your beliefs about how well you performed compared to other participants in this session. In particular, you are part of a group of 8 together with 7 other participants in this session. These 7 participants have been randomly selected by the computer.

We want to know what you think is the probability that your performance is in the top half of performances, that is in the Top 4 of your group of 8 participants. We will then give you some feedback about your performance and ask you again about your belief concerning the probability of scoring in the Top 4 of your group of 8. Apart from the money you can earn with your performance during the exercise, you can earn money for submitting your honest beliefs, as we will explain shortly.

The outline of the experiment is therefore the following:

1. Perform exercise 1, submit your beliefs about your relative performance in exercise 1, receive feedback about relative performance in exercise 1, submit your updated beliefs about your relative performance in exercise 1.

2. Perform exercise 2, submit your beliefs about your relative performance in exercise 2, receive feedback about relative performance in exercise 2, submit your updated beliefs about your relative performance in exercise 2.

3. Perform exercise 3, submit your beliefs about your relative performance in exercise 3, receive feedback about relative performance in exercise 3, submit updated your beliefs about your relative performance in exercise 3.

4. We will explain this part after you completed rounds 1 to 3.

Back

Next

After the experiment the computer will randomly select one of these four rounds, and pay you on the basis of your earnings in that round.

Note that this procedure gives an incentive to perform each exercise and belief elicitation as if it is the one that counts for your payment, as it may indeed be. Also, note that it is not possible to "compensate" earnings in one round with earnings in another round, as only one round will be relevant for payment.

We will now explain in more detail the procedures by which you submit your beliefs and receive feedback.

Back

Next

Belief elicitation

After each exercise, we will ask for your belief regarding the probability that your performance in the exercise is amongst the Top 4 of your group of 8 participants. Your performance is measured in the following way: you receive one point for each correctly solved puzzle and lose half a point for each incorrectly solved puzzle. If there is a tie in the score, the computer randomly chooses which person will rank more highly. In evaluating your relative performance, keep in mind that everybody in your session is a student at the same faculty as you.

The accuracy of your stated probability that you are in the Top 4 of performers will influence your payment. The experiment is designed such that you have the highest chance to earn money if you state the true probability with which you believe that you are in the Top 4.

On the next screen, we will explain how exactly you will be paid for your reported probability.

Back

Next

Belief elicitation

Your stated probability will determine your choice between two options:

•Option A: you receive 10.00 DKK if you are in the Top 4 of performances in your group of 8 participants

•Option B: you receive 10.00 DKK with probability x

x is a number between 0% and 100% and will be randomly picked by the computer. Each number between 0% and 100% is equally likely to be picked.

Your reported probability of being in the Top 4 of performances determines the choice between option A and option B in the following way. If x is higher than your reported probability, option B is chosen and you receive 10.00 DKK with probability x. If x is lower than your reported probability, option A is chosen and you receive 10.00 DKK if you are in the Top 4 of performances.

This means that you always maximize the chances of winning the prize if you state the true probability with which you believe that you are in the Top 4. On the next page we will illustrate this with an example.

Back

Next

For example, let's assume that after performing you believe that it is 62% likely that you are in the Top 4.

•Suppose you report your belief accurately. Then, if *x* is below 62% you will get option A, which gives you a higher probability of winning (namely 62%) than you would get with option B (namely *x*). If *x* is above 62% you will get option B, which gives you a higher chance of winning (namely *x*) than you would get with option A (namely 62%). So, whatever the value of *x*, you will always get the option that gives you the highest chance of winning.

•Suppose you report an inaccurate belief, let's say 10%. Then, if *x* is below 10% you will get option A, which gives you a higher probability of winning (namely 62%) than you would get with option B (namely *x*). If *x* is above 10%, you will get option B, but this option may give you a lower chance of winning than option A. If *x* is between 10% and 62%, you get option B, even though you are better off with option A which gives you a probability of 62%. Reporting a wrong belief will therefore reduce your chances of winning the 10.00 DKK .

The logic in this example holds for all probabilities. Whatever your belief that you are in the Top 4 of your group of eight participants, **you maximize your chance of winning the prize if you report your true belief**.

Reminder

Your stated probability will determine your choice between two options:

•Option A: you receive 10.00 DKK if you are in the Top 4 of performances in your group of 8 participants

•Option B: you receive 10.00 DKK with probability *x*

Back

Next

Control questions

To check your understanding, we now ask you a couple of control questions. The answers to these questions have no influence on your payoffs. Please raise your hand if you have a question and we'll come to your seat and answer it.

To answer the next two questions, assume your true belief is that you are in the Top 4 with a probability of 75%.

Suppose you report your true belief of 75% and the computer picks *x* equal to 90%. What is the probability that you win the prize?

a) 90%   b) 75%   c) 40%   d) 10%

Suppose you report your true belief of 75% and the computer chooses *x* equal to 50%. Would you have had a higher chance of winning by reporting 40% instead of 75%?

a) Higher chance reporting 40%   b) Higher chance reporting 75%   c)The same

Back   Next

Performance feedback

After you have completed the exercise and submitted your belief about your relative performance we give you several rounds of feedback about your relative performance, as we explain below. After each round of feedback we ask again for your belief about your performance.

The feedback on your performance is not precise but contains some noise. The computer will show you a (virtual) ball, which can either be red or black. The ball is drawn from one of two (virtual) boxes. The first box has 7 red balls and 3 black balls. The second box has 3 red balls and 7 black balls. The computer will show you only the color of the ball, not the box it was drawn from. You only know that the relevant box will be selected depending on your performance in the following way.

If your score is in the Top 4, the ball is drawn from the first box, filled with 7 red balls and 3 black balls.

If your score is NOT in the Top 4, the ball is drawn from the second box, filled with 3 red balls and 7 black balls.

This means that a **red ball is a good signal** about your performance while a **black ball is a bad signal** about your performance. But while the color of the ball will give you some information about your performance, you will not learn your performance for sure. The procedure is illustrated in the picture on the next page.

Back

Next

Performance feedback

If your score is in the Top 4, the ball is drawn from the first (left) box, filled with 7 red balls and 3 black balls.

If your score is NOT in the Top 4, the ball is drawn from the second (right) box, filled with 3 red balls and 7 black balls.

This means that a **red ball is a good signal** about your performance while a **black ball is a bad signal** about your performance.

The procedure is illustrated in the picture below:

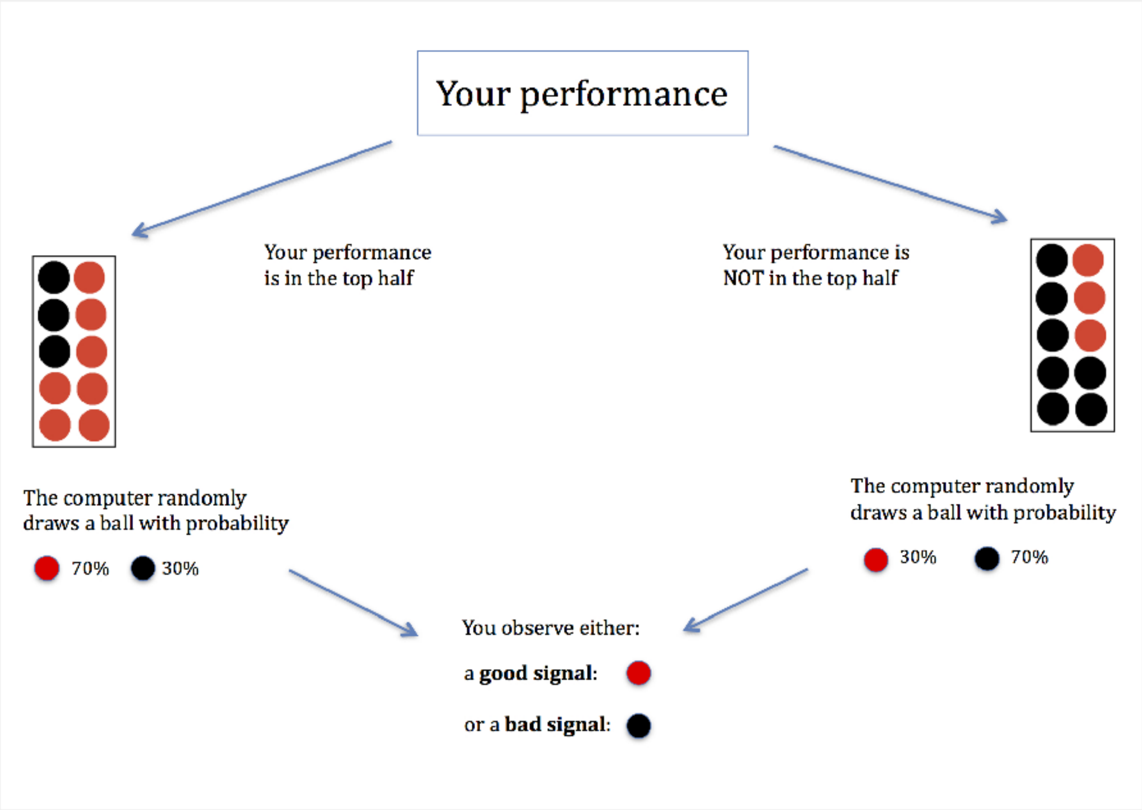

Back

Next

After receiving this feedback, we will ask you to give your updated belief about the probability that you are in the Top 4 of performers of your group of 8 randomly selected participants. This updated belief is elicited in the same way we described above. This means that you can again earn a prize of 10.00 DKK. We repeat this procedure 6 times, so that in total you will see 6 rounds of feedback, i.e. 6 virtual balls. After each round of feedback we ask for your updated belief. In each round, you can earn money depending on the accuracy of your belief, in the way described above. After the last belief elicitation, we move to the next exercise.

Summary

So in summary, the outline for each of the exercises is as follows:

1. Perform the exercise.

2. Submit your initial belief of being in the Top 4 of performers in your group of 8.

3. Receive feedback in the form of a colored ball

4. Submit updated belief.

5. Repeat feedback and belief updating 6 times.

6. Move to the next exercise.

If you have questions about any of the instructions so far, please raise your hand now. Once all questions have been answered, we will proceed to explain the first exercise.

Back

OK

Anagrams

This exercise is designed to measure your ability for languages.

In this exercise you will be shown a series of English words. Your task is to provide one anagram of each word in correct English. An anagram is a word that consists of exactly the same letters as the original word. To construct a correct anagram, you have to use all the letters of the original word.

Example 1: if you are shown the word "felt", a correct solution is the word "left".

Example 2: if you are shown the word "deaf", a correct solution is the word "fade".

You have 5 minutes to complete as many anagrams as you can. If this exercise is selected for payment, you will be paid as follows. You receive 8.00 DKK for each correctly solved anagram minus 4.00 DKK for each incorrectly solved anagram (with a minimum of zero).

Note: only use lower case letters to enter the anagrams; words containing capital letters will always be counted as wrong.

Please raise your hand if you have questions. Otherwise, please click "OK" to start the exercise.

OK

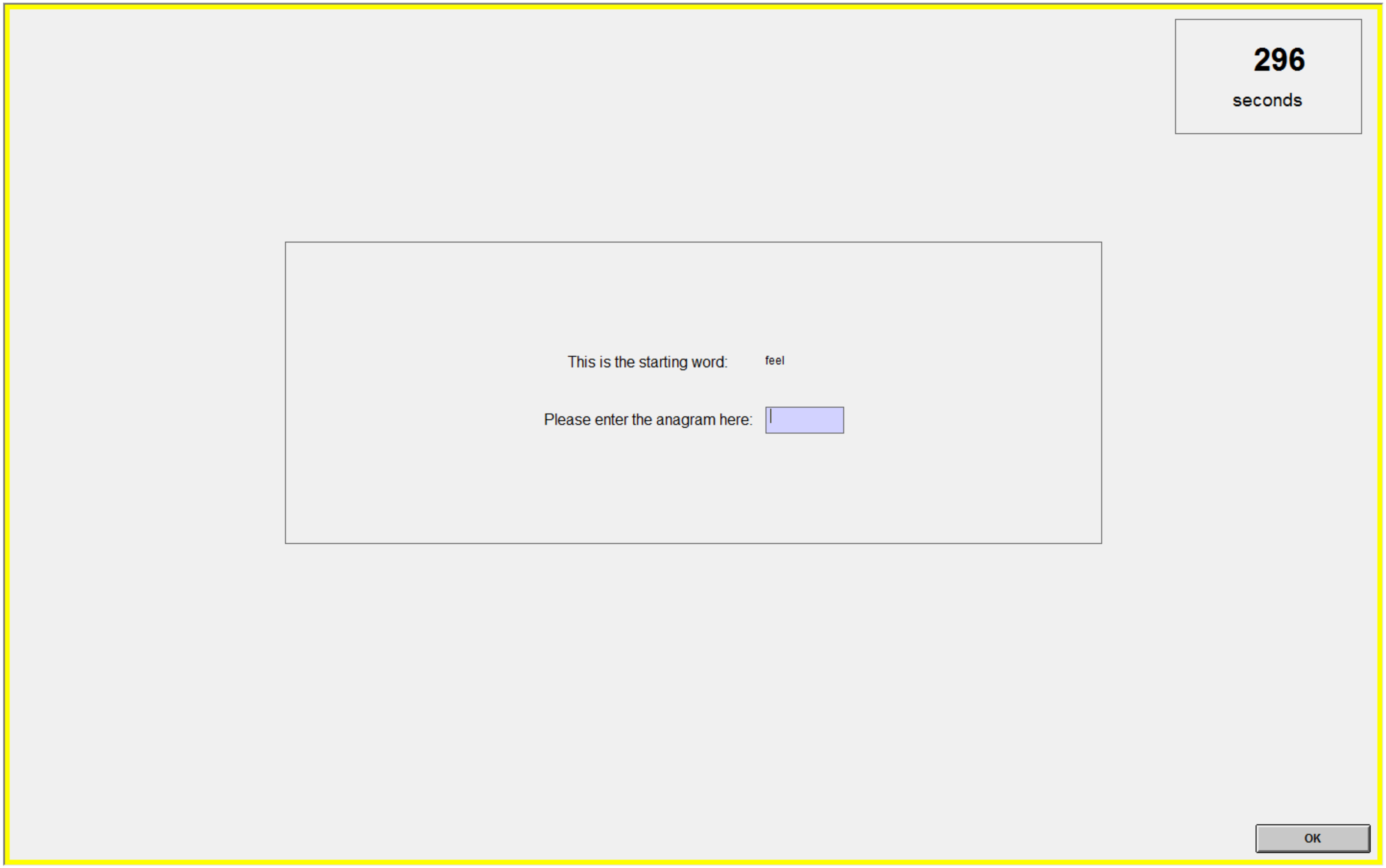
296
seconds
This is the starting word: feel
Please enter the anagram here:
OK

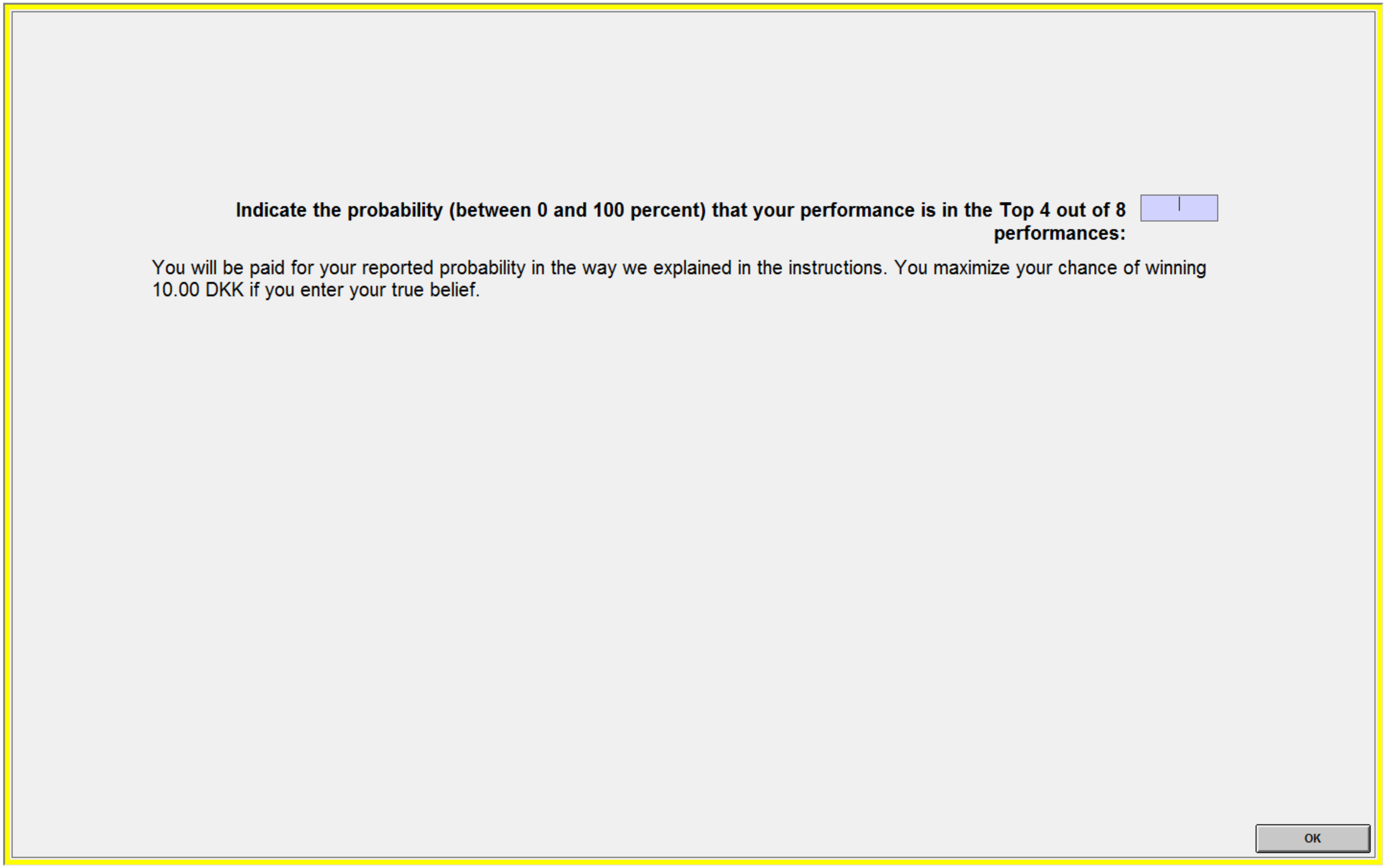

Indicate the probability (between 0 and 100 percent) that your performance is in the Top 4 out of 8 performances:
You will be paid for your reported probability in the way we explained in the instructions. You maximize your chance of winning 10.00 DKK if you enter your true belief.
OK

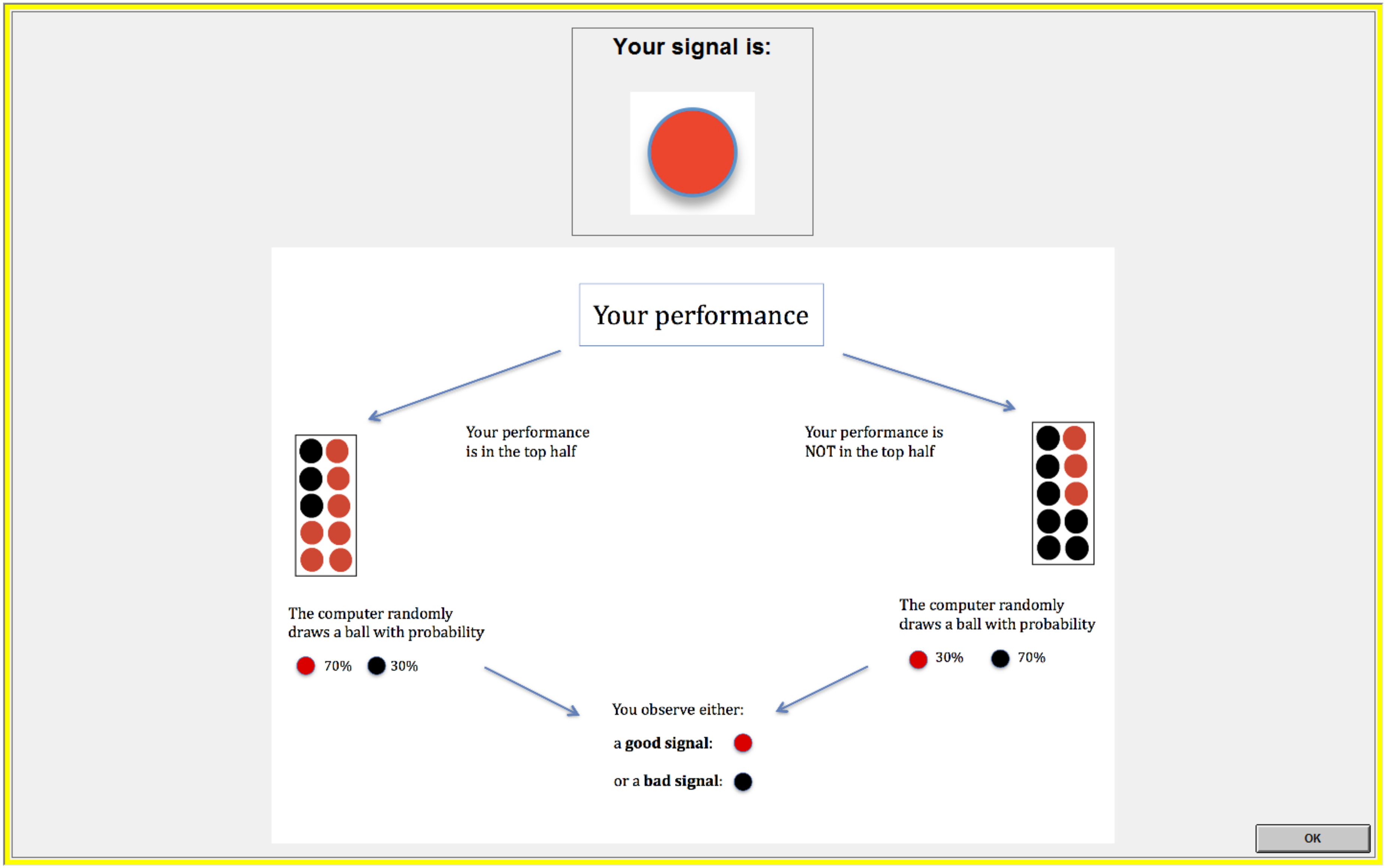
Your signal is:
Your performance
Your performance
is in the top half
Your performance is
NOT in the top half
The computer randomly
draws a ball with probability
70%
30%
The computer randomly
draws a ball with probability
30%
70%
You observe either:
a good signal:
or a bad signal:
OK

Pattern completion

This exercise is designed to measure your general intelligence (IQ).

In this exercise you will be shown patterns with a missing element. Your task is to select from several options given at the bottom of the screen the option that completes the pattern. An example is provided below, where option 2 is the correct answer.

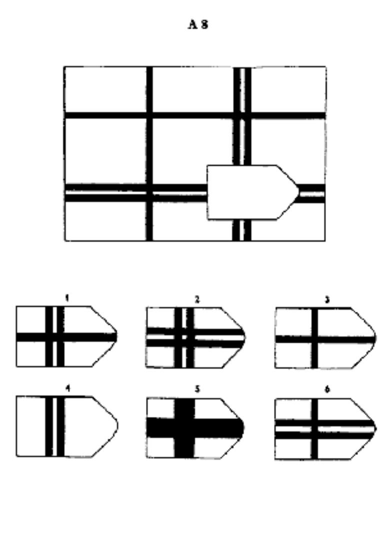

Next

You have 5 minutes to complete as many patterns as you can. If this exercise is selected for payment, you will be paid as follows. You receive 8.00 DKK for each correctly solved pattern minus 4.00 DKK for each incorrectly solved pattern (with a minimum of zero).

Please raise your hand if you have questions. Otherwise, please click "OK" to start the exercise.

Back

OK

295

seconds

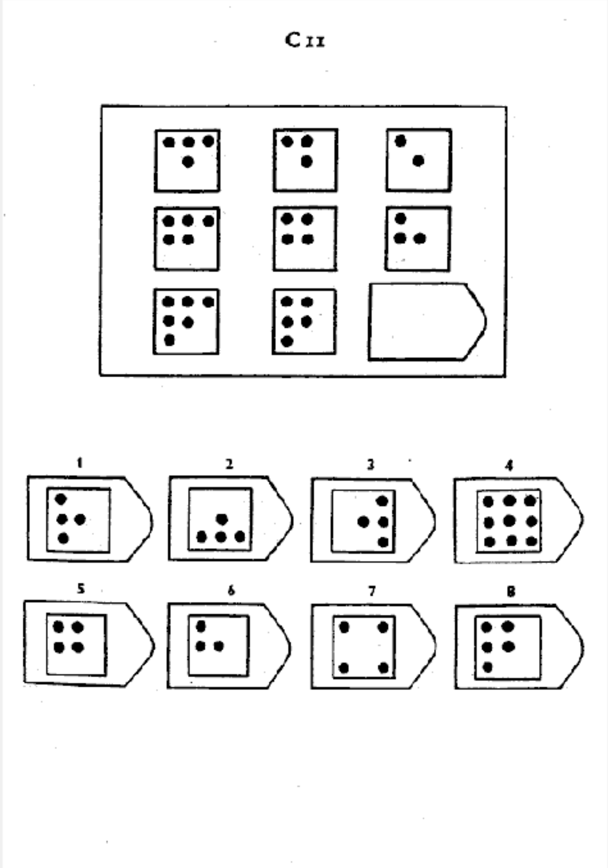

○ 1 ○ 2 ○ 3 ○ 4 ○ 5 ○ 6 ○ 7 ○ 8

OK

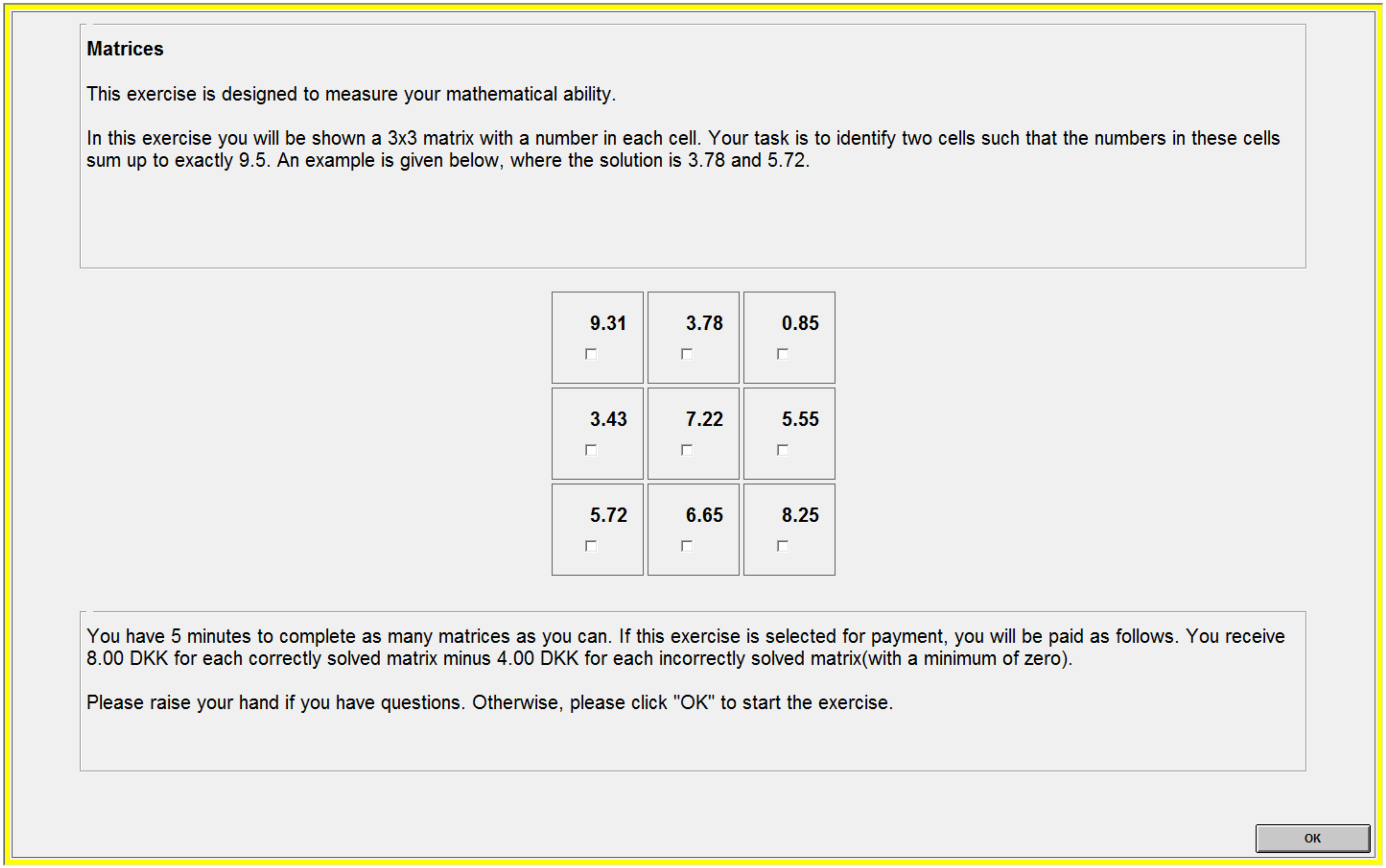
Matrices
This exercise is designed to measure your mathematical ability.
In this exercise you will be shown a 3x3 matrix with a number in each cell. Your task is to identify two cells such that the numbers in these cells sum up to exactly 9.5. An example is given below, where the solution is 3.78 and 5.72.
9.31
3.78
0.85
3.43
7.22
5.55
5.72
6.65
8.25
You have 5 minutes to complete as many matrices as you can. If this exercise is selected for payment, you will be paid as follows. You receive 8.00 DKK for each correctly solved matrix minus 4.00 DKK for each incorrectly solved matrix(with a minimum of zero).
Please raise your hand if you have questions. Otherwise, please click "OK" to start the exercise.
OK

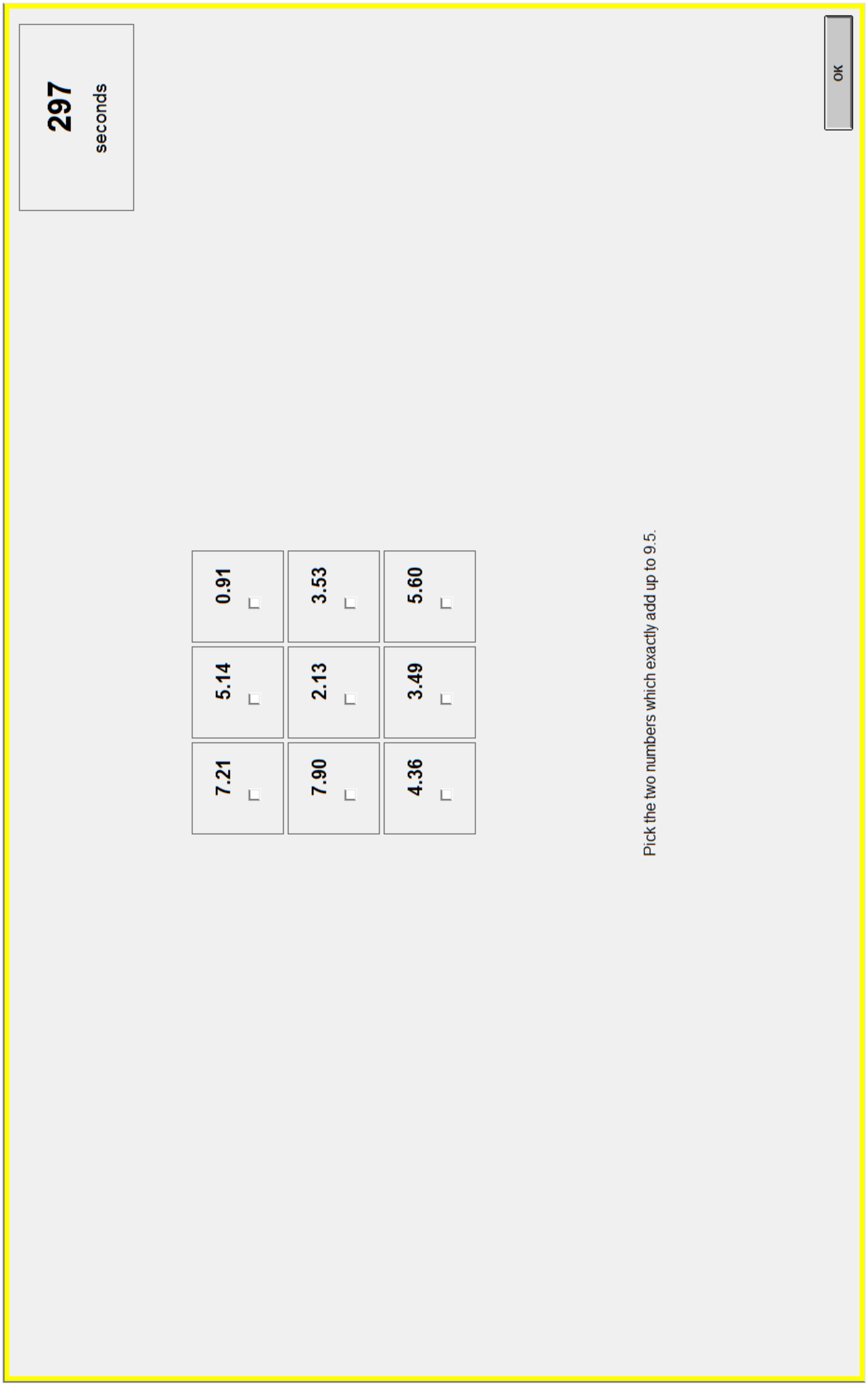
297
seconds
7.21
5.14
0.91
7.90
2.13
3.53
4.36
3.49
5.60
Pick the two numbers which exactly add up to 9.5.
OK

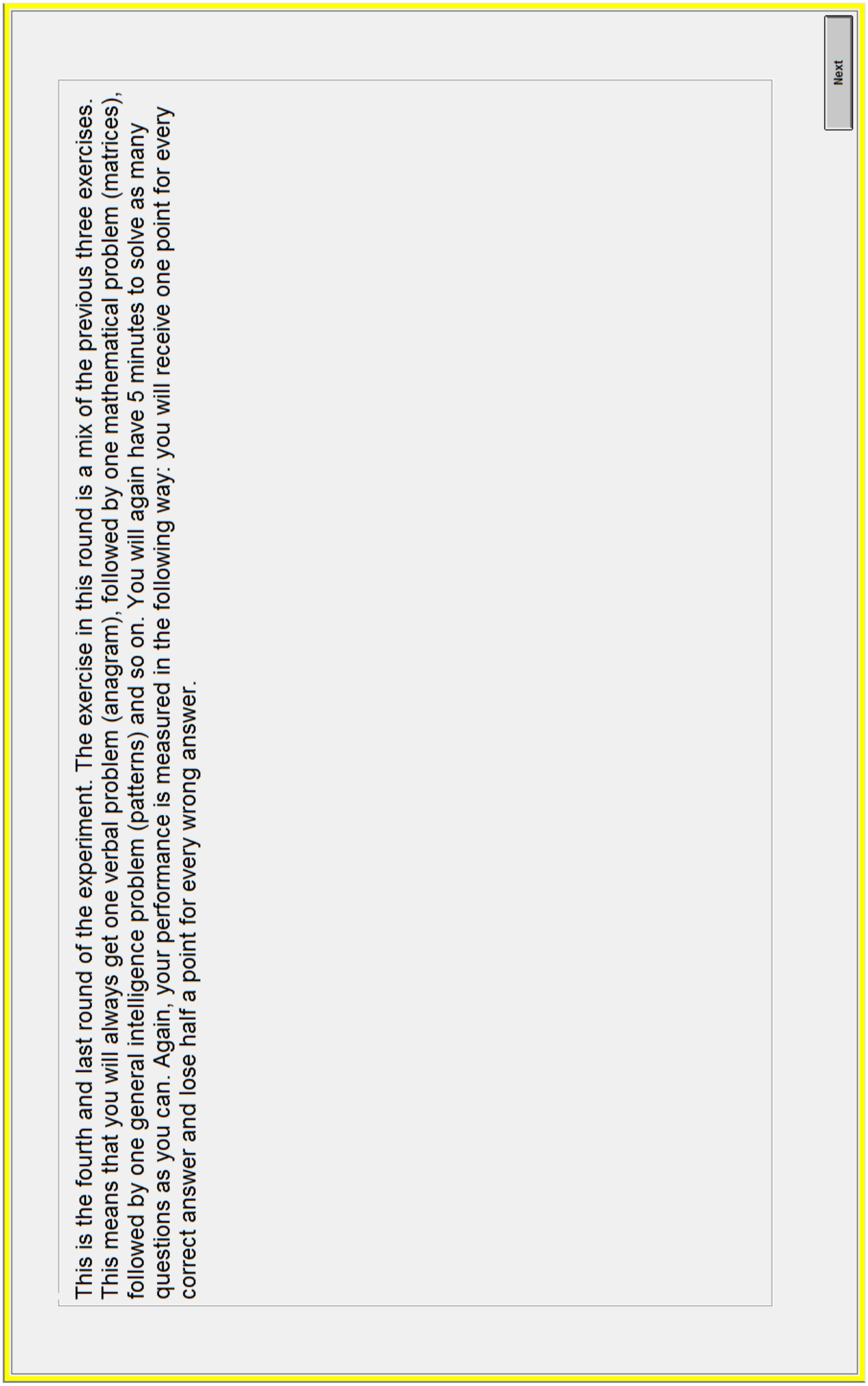
This is the fourth and last round of the experiment. The exercise in this round is a mix of the previous three exercises. This means that you will always get one verbal problem (anagram), followed by one mathematical problem (matrices), followed by one general intelligence problem (patterns) and so on. You will again have 5 minutes to solve as many questions as you can. Again, your performance is measured in the following way: you will receive one point for every correct answer and lose half a point for every wrong answer.
Next

In this round, you will be able to choose how you want to be paid for your performance. Depending on your choice, your payment for this round will depend only on your own performance in the exercise or on your performance compared to the performance of an opponent. This opponent is randomly selected by the computer among the other participants who are in the lab with you.

There will be no belief elicitation in this round.

On the next screen, you will be able to choose how you would like to be paid for your performance in this round. You have the following two options:

1. Piece-rate pay: You receive 12.00 DKK for every point you score in the exercise.

2. Competition pay: You receive 24.00 DKK for every point you score in the exercise if you perform better than your randomly selected opponent and nothing otherwise.

Back

Next

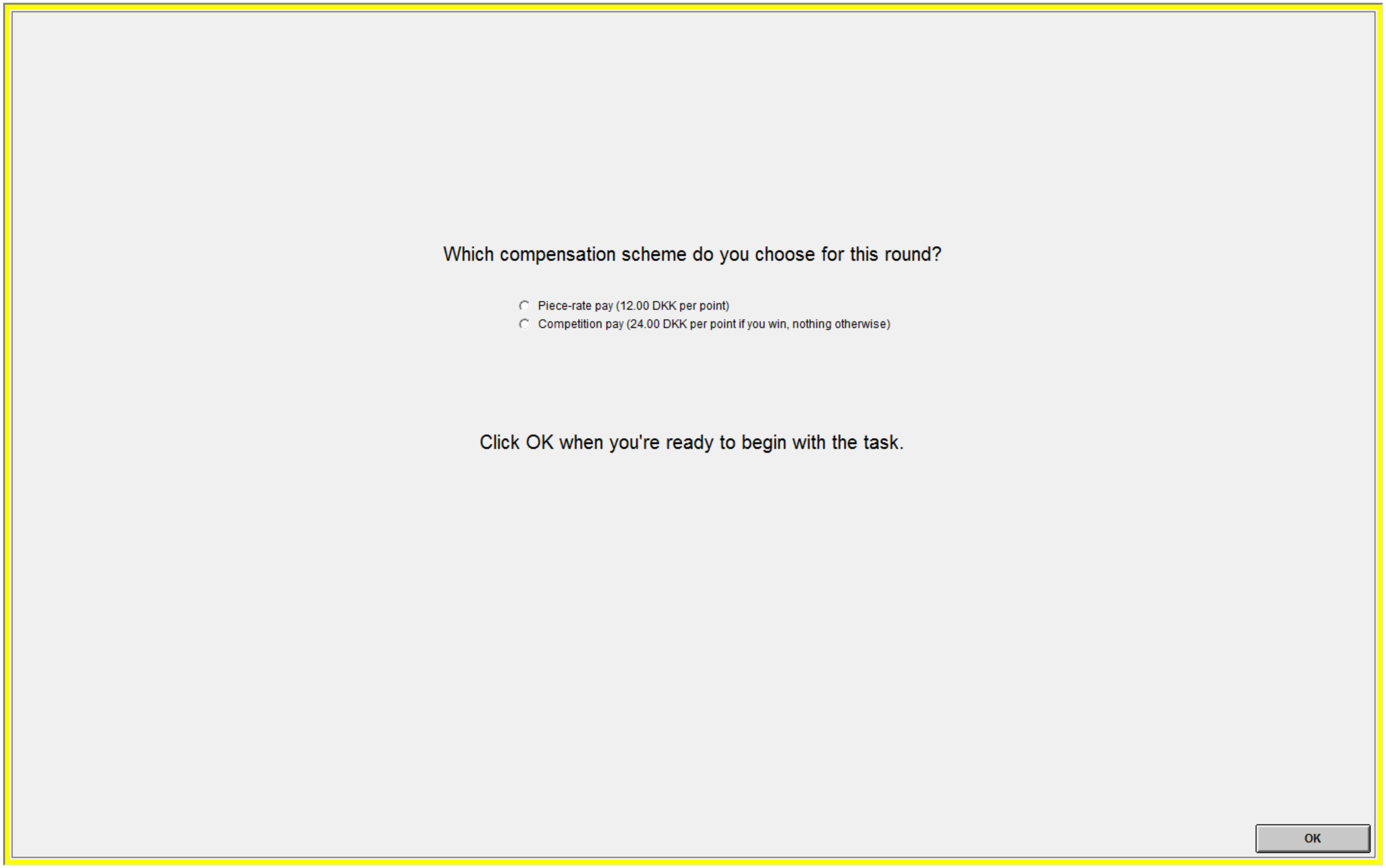
Which compensation scheme do you choose for this round?
Piece-rate pay (12.00 DKK per point)
Competition pay (24.00 DKK per point if you win, nothing otherwise)
Click OK when you're ready to begin with the task.
OK

Before we inform you of your earnings, we would like to ask you a few questions. These questions are important for our research so please take time to answer them.

Please answer the following questions, on a scale from 1 (disagree completely) to 7 (agree completely).

I think the pattern completion problems I solved in this experiment are indicative of the kind of intelligence needed in my field of study.	1	○	○	○	○	○	○	○	7
I think the matrix calculation problems I solved in this experiment are indicative of the kind of intelligence needed in my field of study.	1	○	○	○	○	○	○	○	7
I think the anagrams I solved in this experiment are indicative of the kind of intelligence needed in my field of study.	1	○	○	○	○	○	○	○	7
I put a lot of time and effort in my academic study.	1	○	○	○	○	○	○	○	7
It is important to me that I get good grades in my studies.	1	○	○	○	○	○	○	○	7
I like to talk to friends and relatives about my field of study.	1	○	○	○	○	○	○	○	7
I am considering an academic career.	1	○	○	○	○	○	○	○	7

OK

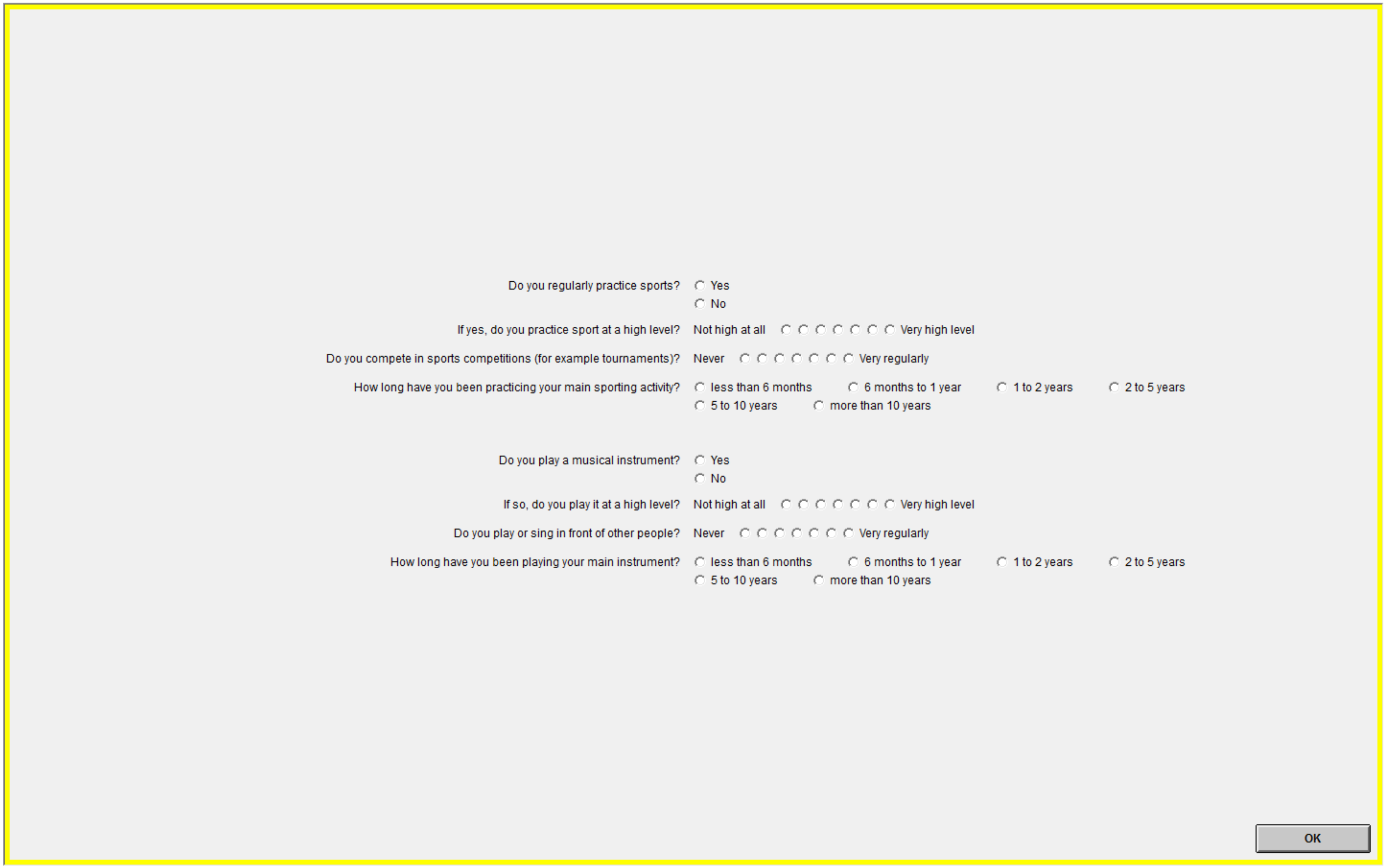
Do you regularly practice sports?
Yes
No
If yes, do you practice sport at a high level?
Not high at all
Very high level
Do you compete in sports competitions (for example tournaments)?
Never
Very regularly
How long have you been practicing your main sporting activity?
less than 6 months
6 months to 1 year
1 to 2 years
2 to 5 years
5 to 10 years
more than 10 years
Do you play a musical instrument?
Yes
No
If so, do you play it at a high level?
Not high at all
Very high level
Do you play or sing in front of other people?
Never
Very regularly
How long have you been playing your main instrument?
less than 6 months
6 months to 1 year
1 to 2 years
2 to 5 years
5 to 10 years
more than 10 years
OK

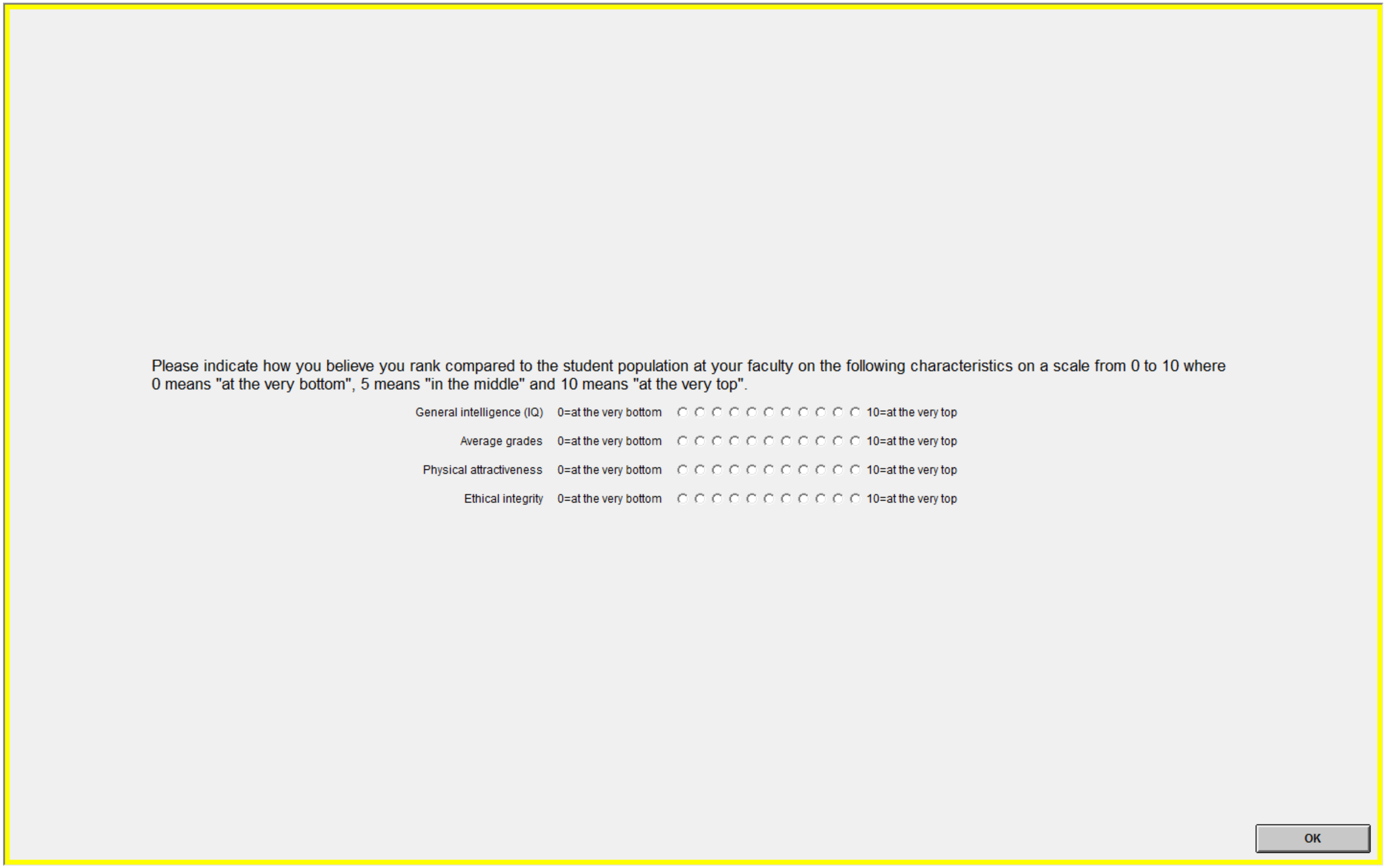
Please indicate how you believe you rank compared to the student population at your faculty on the following characteristics on a scale from 0 to 10 where 0 means "at the very bottom", 5 means "in the middle" and 10 means "at the very top".
General intelligence (IQ) 0=at the very bottom 10=at the very top
Average grades 0=at the very bottom 10=at the very top
Physical attractiveness 0=at the very bottom 10=at the very top
Ethical integrity 0=at the very bottom 10=at the very top
OK

How do you see yourself: Are you generally a person who is fully prepared to take risks or do you try to avoid taking risks?

Please choose a value on the scale below, where the value 0 means "unwilling to take risks" and the value 10 means "fully prepared to take risk".

0=Unwilling to take risks ○ ○ ○ ○ ○ ○ ○ ○ ○ ○ ○ 10=Fully prepared to take risk

How competitive do you consider yourself to be?

Please choose a value on the scale below, where the value 0 means "not competitive at all" and the value 10 means "very competitive".

0=Not competitive at all ○ ○ ○ ○ ○ ○ ○ ○ ○ ○ ○ 10=Very competitive

To what extent do you agree with this statement: *"I am a narcissist."* (Note: The word 'narcissist', means egotistical, self-focused, and vain.)

0=Not at all true of me ○ ○ ○ ○ ○ ○ ○ ○ ○ ○ ○ 10=Very true of me

OK

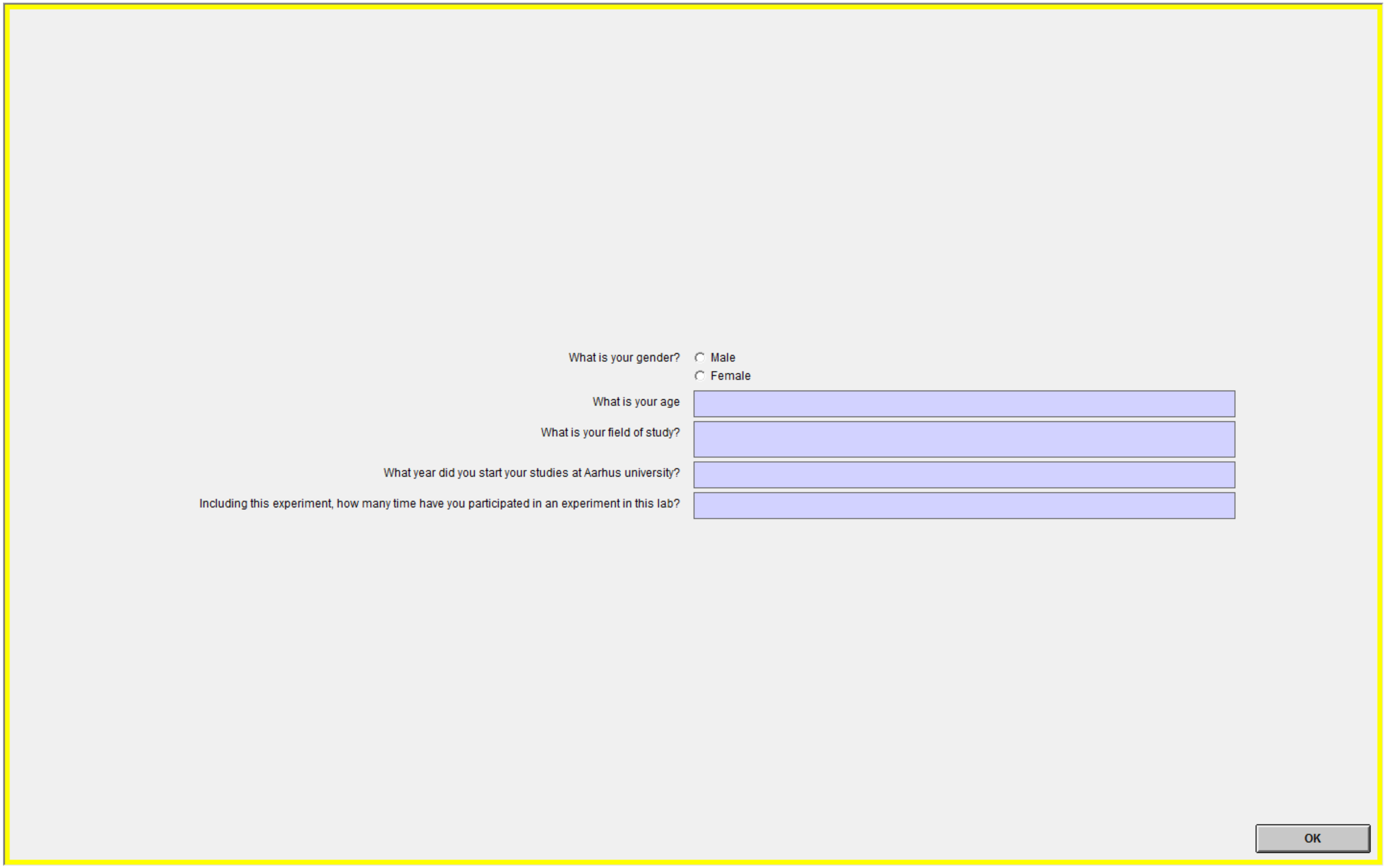

What is your gender? ○ Male ○ Female

What is your age

What is your field of study?

What year did you start your studies at Aarhus university?

Including this experiment, how many time have you participated in an experiment in this lab?

OK

This is the end of the experiment.

The computer randomly chose round 2 for your payment. In that round, you earned 30.00 DKK. This amount and the 40.00 DKK that you received for showing up on time today will be transferred to your NemKonto automatically.

OK
